# Assessment of Fractal Characteristics of Locomotor Activity of Geriatric In-Patients With Alzheimer’s Dementia

**DOI:** 10.3389/fnagi.2019.00272

**Published:** 2019-10-04

**Authors:** Stefan E. Huber, Pierre Sachse, Andreas Mauracher, Josef Marksteiner, Wilfried Pohl, Elisabeth M. Weiss, Markus Canazei

**Affiliations:** ^1^Institute of Ion Physics and Applied Physics, University of Innsbruck, Innsbruck, Austria; ^2^Department of Psychology, University of Innsbruck, Innsbruck, Austria; ^3^Bartenbach GmbH, Aldrans, Austria; ^4^Department of Psychiatry and Psychotherapy A, State Hospital Hall, Hall in Tirol, Austria; ^5^Department of Psychology, University of Graz, Graz, Austria

**Keywords:** locomotor activity, fractal analysis, detrended fluctuation analysis, wrist-actigraphy, fractal scaling, dementia, Alzheimer’s disease

## Abstract

**Introduction:**

Many physiological signals yield fractal characteristics, i.e., finer details at higher magnifications resemble details of the whole. Evidence has been accumulating that such fractal scaling is basically a consequence of interaction-dominant feedback mechanisms that cooperatively generate those signals. Neurodegenerative diseases provide a natural framework to evaluate this paradigm when this cooperative function declines. However, methodological issues need to be cautiously taken into account in order to be able to provide reliable as well as valid interpretations of such signal analyses.

**Methods:**

Two conceptually different fractal analyses, i.e., detrended fluctuation analysis (DFA) and analysis of cumulative distributions of durations (CDDs), are applied to actigraphy data of 36 geriatric in-patients diagnosed with dementia. The impact of the used time resolution for data acquisition on the assessed fractal outcome parameters is particularly investigated. Moreover, associations between these parameters and scores from the Mini-Mental-State-Examination and circadian activity parameters are explored.

**Results:**

Both analyses yield significant deviations from (mono-)fractal scaling over the entire considered time range. DFA provides robust measures for the observed break-down of fractal scaling. In contrast, analysis of CDDs results in measures which highly fluctuate with respect to the time resolution of the assessed data which affects also further derived quantities such as scaling exponents or associations with other (clinically relevant) assessed parameters.

**Discussion:**

To scrutinize actigraphic signal characteristics and especially their (deviations from) fractal scaling may be a useful tool for aiding diagnosis, characterization, and monitoring of dementia. However, results may, besides contextual aspects, also substantially depend on specific methodological choices. In order to arrive at both reliable and valid interpretations, these complications need to be carefully elaborated in future research.

## Introduction

It has been recognized in the cognitive sciences that multi-component systems with a high degree of interaction and feedback give rise to emergent signals which yield fractal scaling indicating self-similarity or rather self-affinity in the case of time series data (Holden et al., 2009; Kello et al., 2010). That means that they exhibit surface-structural features which remain (statistically) invariant over various (e.g., temporal or spatial) scales. Since the functional system governing (human) motor control represents a pivotal example for such a system (Klix, 1971; Grafton et al., 2009; Hacker and Sachse, 2014), it may not come as a surprise that a variety of physiological signals regulated by the central nervous system such as heart rate (Costa et al., 2002; Varotsos et al., 2007, 2019), respiration rate (Peng et al., 2002), speech (particularly, acoustic fluctuations in articulation; Kello et al., 2008), gait (Buzzi et al., 2003) and prolonged, unconstrained standing (Duarte and Zatsiorsky, 2001) among many others (see e.g., McGrath, 2016) exhibit fractal characteristics. This characteristics has become recognized as an earmark of healthy physiology being closely related to an organism’s ability to actively maintain optimal levels of adaptability and flexibility under variable (external) conditions (West and Deering, 1994; Van Orden et al., 2003, 2005; West, 2009, 2010; McGrath, 2016). For instance, in the case of electrocardiograms, the break-down of the long range temporal correlations, i.e., the fractal scaling, which characterizes healthy heart rate variability, was found to be associated with high risk of sudden cardiac death (Varotsos et al., 2007, 2019). This notion of health challenges the traditional theory of homeostasis (West and Deering, 1994; Van Orden et al., 2005; West, 2009, 2010) as it ascribes the healthy organism an active role in balancing exploratory versus sustaining behavior. Also human locomotor activity assessed by wrist-actigraphy has repeatedly been found to yield fractal regulation (Hu et al., 2004, 2009, 2013, 2016; Ohashi et al., 2004; Nakamura et al., 2007, 2013a, 2016; Paraschiv-Ionescu et al., 2008; Aybek et al., 2012; Sano et al., 2012).

Detrended fluctuation analysis (DFA) has been repeatedly applied to activity data of subjects with dementia. DFA quantifies the magnitude of activity fluctuations as a function of time scale (Hu et al., 2004, 2009, 2013, 2016; Li et al., 2018). If this function takes the form of a power law characterized by a parameter known as scaling exponent, see section “Detrended Fluctuation Analysis,” the fluctuations yield fractal scaling, i.e., their magnitude scales with the temporal unit used for their quantification. Moreover, the scaling exponent is an indicator for temporal correlations present in the signal. Whereas scaling exponents of 0.5 are obtained for uncorrelated noise, scaling exponents exceeding 0.5 indicate positive correlations, for which large (small) fluctuations are more likely to be followed by large (small) fluctuations. Scaling exponents of 1.5 are indicative of Brownian motion (also known as red noise) exhibiting a high degree of said regularity. Interestingly, scaling exponents for healthy control subjects are close to 1, in particular, ∼ 0.8–1.1 (Hu et al., 2004; Ohashi et al., 2004; Paraschiv-Ionescu et al., 2008; Aybek et al., 2012). In addition, they were found to be independent of individual average activity levels and circadian phase and are unlikely to be a consequence of random or scheduled events, but rather indicate an underlying mechanism of motor control with stable fractal characteristics over time scales ranging from minutes to hours (Hu et al., 2004). Moreover, it was shown that elderly subjects with dementia were associated with disrupted fractal scaling, i.e., activity fluctuations could not be described by a single power law over the entire time range. In contrast, two regions, below ∼1.5 h and above ∼2 h, with distinct characteristic scaling exponents were identified (Hu et al., 2009). The scaling exponent for the longer time ranges was correlated with the age and diagnosis of dementia (Hu et al., 2009), whereas a decreasing scaling exponent at shorter time scales was associated with the progression of cognitive decline (Hu et al., 2016). Furthermore, the difference between the two scaling exponents, i.e., a measure for the break-down of fractal scaling over the entire time range, has been proposed as a (non-invasive) biomarker of the degeneration of the suprachiasmatic nucleus in dementia (Hu et al., 2013). In addition, this difference was shown to be more pronounced in patients with more amyloid plaques (Hu et al., 2013), which is positively correlated with the severity of Alzheimer’s disease (AD).

The analysis of cumulative distributions of durations (CDDs), another variant of fractal analysis, aims at characterizing how periods of high and low locomotor activity are distributed over time (Nakamura et al., 2007; Paraschiv-Ionescu et al., 2008). Interestingly, also the analysis of CDDs was reported to yield fractal scaling of the complementary, cumulative distributions of low-activity period durations. Its characteristic scaling exponent was shown to be close to 1 for healthy control subjects (Nakamura et al., 2007, 2008, 2013a,b, 2016; Sano et al., 2012). Moreover, the power law form of the distributions appeared to be preserved also in cases of various psychopathological syndromes, however, each of them being associated with characteristic scaling exponents significantly different from 1 (Nakamura et al., 2007, 2013a, 2016; Sano et al., 2012). To our best knowledge, analysis of CDDs has so far not been applied to actigraphic data of subjects with dementia. As dementia affects locomotor activity (Cummings, 1997; American Psychiatric Association, 2013), one could hypothesize that this might be reflected also in characteristics of corresponding CDDs. Moreover, one could further hypothesize that in the case of neurodegeneration, also an analysis of CDDs should result in distributions of low-activity period durations that deviate from power law form, indicating thus a break-down of fractal scaling, as it has been found in the case of DFA.

For these reasons, we applied both DFA and an analysis of CDDs to actigraphy data of geriatric in-patients diagnosed with dementia. We subsequently computed several measures in order to quantify the extent of deviation from fractal scaling. We also explored associations between those measures and age, circadian activity parameters and scores obtained from the Mini-Mental-State-Examination (MMSE; Folstein et al., 1975; Kessler et al., 1990). Finally, we investigated how sensitive the several outcomes are to the time resolution of the analyzed activity signals in order to explore their reliability with respect to this parameter.

Due to largely lacking data which could clinically and psycho-pathologically characterize the sample and the high extent of heterogeneity in terms of comorbidities and interventions including especially medication which are typical complications in studies involving AD, we would like to emphasize already at this stage that we explicitly do not regard this investigation in any way to provide a stringent test of the hypothesis that AD as such represents a specific cause for a general loss of fractal regulation of human motor activity. Evidence is accumulating corroborating such an expectation, though (Hu et al., 2009, 2013, 2016; Li et al., 2018). However, here, we rather aim to explore some of the capabilities of the considered non-linear methodology when it is applied in a setting typically faced in clinical reality in the case of AD. In particular, we ask which conceptually expectable implications may robustly remain in spite of the high degree of heterogeneity in the data. Moreover, we particularly focus on technical and methodological issues which are nevertheless required to be considered in future research activities aiming at more rigorous tests of the above mentioned hypothesis and its implications.

## Materials and Methods

### Subjects and Wrist Actigraphy

Patients were recruited at the Department of Psychiatry and Psychotherapy A of the State Hospital Hall in Tirol, Austria. All patients were examined according to a standardized protocol. This examination included a clinical examination, a review of medical records, laboratory testing, cognitive testing and neuroimaging. Patients were included in the study when they met the inclusion criteria. The main inclusion criterion was the presence of Alzheimer’s dementia [F00 according to the ICD-10 classification of mental and behavioral disorders (World Health Organization [WHO], 1992)]. Patients for who (i) diagnoses of other mental or behavioral disorders or other types of dementia (except for delirium superimposed on dementia; F05.1 according to the ICD-10) were listed in their records (provided by the hospital personnel) and/or patients who (ii) removed their actigraphs autonomously before the end of their hospital stays were excluded. The resulting sample comprised actigraphic data of 36 patients (19 women and 17 men; 61–94 years old; mean [SD] age: 81.8 [7.8] years). Additional diseases of those patients are listed in Table 1 and more detailed information is provided in Section 1 of the Supplementary Material accompanying this work. Three participants showed day-night reversal. 16 participants were autonomously mobile, 17 needed a walking aid or wheel chair and thus had limited mobility and for three participants no information on their mobility was recorded. The participants’ cognitive function was assessed with the MMSE (Folstein et al., 1975; Kessler et al., 1990); mean score (SD): 13 (7). The study was approved by the ethical committee of the Medical University of Innsbruck.

**TABLE 1:** Characteristics of the sample (mean with SD or number of subjects with percentage in parenthesis).

Age	81.8(7.8)
Gender, *n* females	19(53%)
MMSE score^a^	13(7)
Length of actigraphic recordings in days	19.7(6.2)
Average physical activity in CPM^b^	99(89)
Mobility^c^, *n* autonomously mobile subjects	16(44%)
Other diagnoses^d^	I10: Essential (primary) hypertension (15; 42%); N39.0: Urinary tract infection (14; 39%); E53.8: Deficiency of other specified B group vitamins (8; 22%); E87.6: Hypokalemia (7; 19%); I25.1: Atherosclerotic heart disease of native coronary artery (6; 17%); G45.9: Transient cerebral ischemic attack (5; 14%); J44.9: Chronic obstructive pulmonary disease (4; 11%); E03.9: Hypothyroidism (4; 11%); N18.9: Chronic kidney disease (4; 11%); K59.0: Constipation (4; 11%)

*^*a*^Available for only 31 of 36 subjects; ^*b*^Quantified as the mean of M10 parameters; ^*c*^For three subjects (8%) no information on their mobility recorded; ^*d*^Other than mental or behavioral disorders: only the ten most abundant are listed, a more comprehensive list of diagnosed comorbidities is provided in the Supplementary Material.*

After written informed consent was obtained an accelerometer of the type ActiGraph wGT3X-BT (Pensacola, FL, United States) was attached to each participant’s wrist during the first two in-patient treatment days in order to be henceforward continuously worn. A fabric lock wristband deterred removal of the actigraph. The accelerometers recorded accelerations experienced at the location of the devices with respect to a three-dimensional device-specific coordinate system using a sampling frequency of ν = 30 Hz. Subsequently, we computed the magnitudes of these raw, three-dimensional accelerations and corrected them for the influence of gravitational acceleration g = 9.81 m/s^2^ by subtracting the latter from them. The magnitudes were then converted to a binary signal yielding a value of 1 if the acceleration magnitude exceeded 0.1 g and 0 otherwise. These binary values were then summed over non-overlapping time intervals each containing τ time points with τ = νδ and δ denoting the length of the time intervals in seconds. In order to investigate the dependence of our results on temporal resolution we used δ = 5, 10, 15, 30, and 60 s yielding counts (i.e., how often the acceleration magnitudes exceed the threshold of 0.1 g) per every 5, 10, 15, 30, and 60 s, respectively. For the remainder of this work, we refer to these data for brevity as CP5s, CP10s, CP15s, CP30s, and CPM which is short for counts per 5, 10, 15, 30 s and for counts per minute (we stick to this different notation in the case of 60 s = 1 min intervals for the frequent use of this short notation CPM in the literature), respectively. On average (SD) 19.7 (6.2) days of actigraphic recordings were analyzed, ranging from 11.6 to 39.6 days.

### Fractal Analyses

#### Analysis of (Complementary) Cumulative Distributions of Durations

**FIGURE 1:**
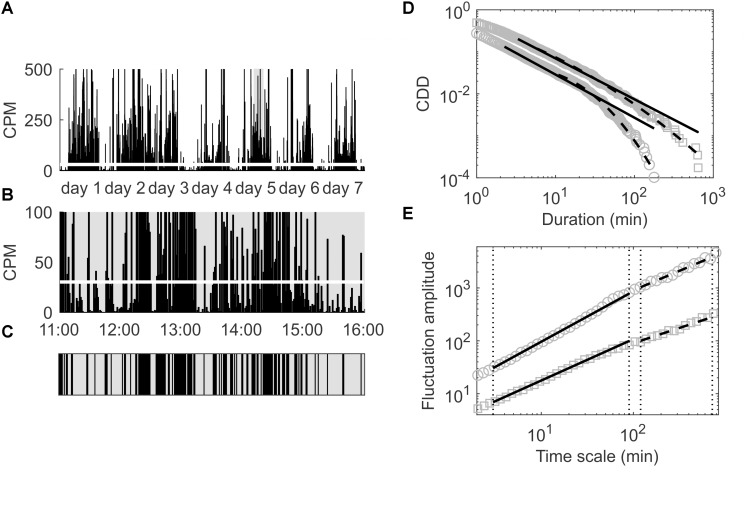
**(A)** Locomotor activity in CPM for the first seven consecutive days of a patient included in the present study; the overall mean activity is indicated by the thick white line. **(B)** Magnification of the time span of 11:00–16:00 at day 5 to illustrate the process of dichotomization of the data in order to analyze the empirical CDD of this patient. **(C)** Dichotomized data corresponding to the time span shown in **(B)**; black areas represent durations during which the activity is above the overall average (thick white line in **B**), gray areas represent durations during which the activity is below that threshold. **(D)** Empirical CDD of the same patient (gray squares) and of another patient (gray circles) for comparison; distribution functions of best fitting power law (black solid lines) and best fitting lognormal distributions (black dashed lines) are also shown. **(E)** Fluctuation amplitudes for the same patients as in **(D)** versus time scale in minutes; short (below 1.5 h) and long (beyond 2 h) time ranges are indicated by vertical dotted lines and arrows; fitted power laws in those two time ranges are depicted using solid and dashed black lines, respectively.

For the analysis of the cumulative distribution of low-activity period durations (CDDs) the time series of accumulated activity counts (i.e., the CP5s, CP10s, CP15s, CP30s, or CPM, see section “Subjects and Wrist Actigraphy”) of each participant was dichotomized into periods yielding activities lower and higher than the overall mean activity, see also Figure 1. In particular, the overall mean activity in terms of the CP5s, CP10s, CP15s, CP30s, and CPM activity data of each participant was calculated first (see also Figures 1A,B). Subsequently, the activity data was decomposed into disjunct intervals in which the activity was lower or higher than the overall mean activity (Figure 1C), i.e., it was decomposed into low- and high-activity periods. Then, the durations of the low-activity periods were determined and sorted from shortest to longest, i.e., *d*_1_ < *d*_2_ < … < *d*_*n*_ with *d*_*i*_ denoting the *i*-th shortest low-activity period duration and *d*_*1*_ and *d*_*n*_ denoting the shortest and longest of all low-activity period durations determined for the respective participant. The complementary cumulative distribution (CDD), *P*(*X*≥*d*), i.e., the probability to find a low-activity period of a duration longer than or equal to *d*, was then calculated for each patient according to:

$$P\left(X\geq d\right)\equiv \int_{d}^{\infty }p\left(x\right)dx\approx \frac{1}{N}\sum_{i=n_{d}}^{n}N_{i}\left(d_{i}\right)$$

Here, the probability density function *p*(*x*) of low-activity periods with durations between *x* and *x+dx* is approximated with the empirical frequency of low-activity period durations *d*_*i*_, i.e., the ratio *N*_*i*_(*d*_*i*_)/*N* with $N=\sum_{i=1}^{n}N_{i}\,\left(d_{i}\right)$, *N*_*i*_(*d*_*i*_) denoting the number of low-activity periods with duration *d*_*i*_, and *n*_*d*_ denoting the index of the shortest determined duration equal or longer than *d* in the sorted set of low-activity period durations described above.

Cumulative distributions of durations resembling a power law, i.e., *P*(*X*≥*d*)∝*d*^−γ^ with γ > 0 denoting the scaling exponent, suggest fractal scaling and have been observed for low-activity periods obtained repeatedly via the same procedure of dichotomization in earlier investigations (Nakamura et al., 2007, 2008, 2013a,b, 2016; Sano et al., 2012). However, these studies did not use actigraphy data from patients with dementia, whereas in the present work, we particularly investigated CDDs of low-activity durations for participants with dementia. In order to quantify deviations of the resulting CDDs from power law form, we fitted both a power law and the CDD of a lognormal distribution to the CDDs via maximum-likelihood (Clauset et al., 2009) building on the procedure described by Mirman et al. (2012).

In particular, the probability density function for a power law reads:

$$p^{\text{PL}}\,\left(x\right)=c_{\text{PL}}\,x^{-\text{β}},c_{\text{PL}}=\left(\text{β}-1\right)\,d_{\text{min}}^{\beta -1}$$

with the fitting parameters β = γ + 1 (with the scaling exponent γ introduced above) and *d*_*min*_. The probability density function for a lognormal distribution reads:

$$p^{\text{LN}}\,\left(x\right)=c_{\text{LN}}\,\frac{1}{x}\,\exp\left[-\frac{{\left(\text{ln}\,\left(x\right)-\text{μ}\right)}^{2}}{2\,\sigma^{2}}\right],$$

$$c_{\text{LN}}=\sqrt{\frac{2}{\pi \,\sigma^{2}}}\,{\left[\text{erfc}\,\left(\frac{\text{ln}\,\left(d_{\text{min}}\right)-\text{μ}}{\sqrt{2}\,\sigma }\right)\right]}^{-1},$$

with fitting parameters µ, σ, and *d*_*min*_. The best fits to the empirically determined durations of low-activity periods, i.e., the {*d*_*i*_}*i* = 1,…,*n* introduced above, are then obtained via maximum-likelihood, i.e., by finding fitting parameters such that the likelihoods given specific values for the fitting parameters:

$$p\,\left({\left\{d_{i}\right\}}_{i=1,\ldots ,n}|\beta ,d_{\text{min}}\right)=\prod_{i=1}^{n}p^{P\,L}\,\left(d_{i}\right)$$

and

$$p\,\left({\left\{d_{i}\right\}}_{i=1,\ldots ,n}|\text{μ},\sigma ,d_{\text{min}}\right)=\prod_{i=1}^{n}p^{\text{LN}}\,\left(d_{i}\right)$$

become maximal for the power law and the lognormal distribution, respectively.

Note that both probability density functions include a lower bound of durations, *d*_*min*_, such that $\int_{d_{\text{min}}}^{\infty }p\,\left(x\right)\,d\,x=1$. The lower bounds for the power law and the lognormal distribution that provide the best fits to a given CDD are usually not equal, see also Figure 1. Since deviations from power law form were quantified by assessing the log-likelihood-ratio (LLR) of the obtained likelihoods for the two distributions (Vuong, 1989), this was done pair-wise and subsequently: first, $d_{\text{min}}=d_{\text{min}}^{\text{PL}}$ was computed for a power law and then also a lognormal distribution was fitted using the same *d*_*min*_ and finally the two obtained fits were compared via the LLR; second, the procedure was repeated analogously for $d_{\text{min}}=d_{\text{min}}^{\text{LN}}$ computed first via fitting a lognormal distribution to the data and then a power law using that *d*_*min*_. As an alternative measure of the deviation from power law form we computed the ratios of goodness-of-fit (GOF) obtained via the Kolmogorov–Smirnov-distance metric (Clauset et al., 2009) for the best fitting power law and lognormal distributions each corresponding to its own optimized minimum durations, i.e., $d_{\text{min}}^{\text{PL}}$ or $d_{\text{min}}^{\text{LN}}$, respectively.

All computations were performed using the software R (R Development Core Team, 2017). For fitting power law and lognormal distributions to the CDDs and computing their LLRs the poweRlaw package was used (Gillespie, 2015).

#### Detrended Fluctuation Analysis

Detrended fluctuation analysis has been described in detail elsewhere (Peng et al., 1995, 2002). In short, the actigraphic signal is first centered (with respect to its mean), integrated and decomposed into disjoint time windows of length *m*. Subsequently for each of those windows, the root-mean-square residuals from a trend fitted to the integrated signal in each window are computed. Taking the average of the residuals over all windows of a given size and repeating the procedure for various window sizes then yields the fluctuation *F*(*m*) as a function of the time scale *m*. Again, a power law functional form, i.e., *F*(*m*)∝*m*^α^, indicates fractal scaling with the scaling exponent α > 0. We separately calculated two scaling exponents in distinct time ranges, in particular α_*1*_ for time scales below 1.5 h and α_*2*_ for time scales beyond 2 h as done in earlier work (Hu et al., 2009), see also Figure 1. The difference between those two exponents, α_12_ = α_1_−α_2_, and its absolute value, |α_12_|, were used to quantify the deviation of the fluctuation function from a power law over the whole time range for which fractal scaling was observed for various physiological outputs under healthy conditions (Hu et al., 2004; Ohashi et al., 2004; Paraschiv-Ionescu et al., 2008; Aybek et al., 2012). All computations were performed using the software R (R Development Core Team, 2017) and employing the dfa function (Penzel et al., 2003) provided in the R package non-linear T series (Garcia, 2015). In particular, we used 66 window sizes ranging from 3 to 720 min, whereas the entire time range has been split into two distinct ranges from 3 to 90 and from 120 to 720 min for calculating the two scaling exponents α_*1*_ and α_*2*_ as outlined above.

### Assessment of Circadian Parameters

Dementia and especially AD is associated with circadian activity rhythm disturbances (Ancoli-Israel et al., 2002; Hatfield et al., 2004) which can be quantified with a non-parametric analysis of actigraphy data (Van Someren et al., 1999). Here, we assessed the following parameters: (a) inter-daily stability (IS) which provides a measure for the stability of locomotor activity across days, (b) intra-daily variability (IV) which provides a measure for the fragmentation of the rest-activity rhythm, (c) rhythm amplitude (RA) quantifying the average span of activities between the ten most active and five most inactive hours in a day, (d) averages of activity of the ten consecutive hours with maximal activity (M10). For computation of these parameters we used the routines implemented in the R software package nparACT (Van Someren et al., 1999; Blume et al., 2016).

### Statistical Analyses

For all computed means of outcome parameters which indicate a deviation from fractal scaling (i.e., |α_12_|, LLR and GOF ratios) bias-corrected and accelerated bootstrap 95%-confidence intervals (Efron, 2012) were computed and interpreted regarding statistical significance of the results. The same was done for the Pearson correlation coefficients computed in order to explore associations between DFA parameters, the MMSE scores, circadian activity parameters and the age of the study participants. For comparison of power law and lognormal fits in the case of individual CDDs, *p*-values were computed according to Vuong’s method (Vuong, 1989). In this case, we have taken *p* < 0.1 as a criterion that the sign of the LLR is a reliable indicator for which distribution provides the better fit to the respective CDD (Clauset et al., 2009).

## Results

### Assessment of Deviations From Fractal Characteristics

**FIGURE 2:**
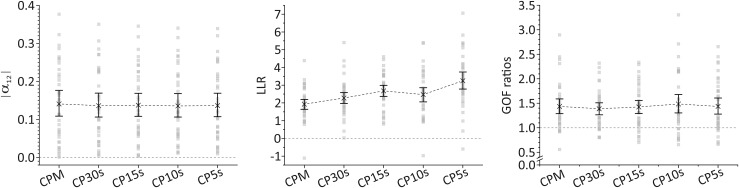
Sample means and bootstrapped 95%-CIs for the absolute values of the difference between scaling exponents for short (below 1.5 h) and long (beyond 2 h) time ranges as obtained via DFA, |α_12_| **(left panel)**, for the log-likelihood-ratios of the computed likelihood that a CDD results from a lognormal distribution relative to a power law distribution, LLR **(middle panel)**, and for goodness-of-fit (GOF) ratios computed using the Kolmogorov–Smirnov distance metric for evaluating the GOFs for lognormal and power law distributions **(right panel)** for all considered time resolutions. Threshold values indicating the deviation from fractal scaling are shown for all three cases in form of dashed, horizontal lines (at 0 for |α_12_| and LLR and at 1 for GOF ratios). For LLR, a positive sign indicates that a lognormal distribution fits the data better than a power law distribution. For GOF ratios, a value larger than 1 indicates that a lognormal distribution fits the data better than a power law distribution.

The assessed measures for deviations from fractal scaling (i.e., |α_12_| for fluctuation amplitudes determined via DFA and LLR as well as GOF ratios for analysis of CDDs) result in a significant deviation of sample means from the respective values associated with fractal scaling irrespective of the used time resolution of the activity data, see Figure 2. In particular, sample means of |α_12_| range from 0.141 with 95%-CI [0.109, 0.177] for the lowest time resolution of CPM to 0.136 with 95%-CI [0.106, 0.169] for the highest time resolution of CP5s. A complete list for all time resolutions is supplied in the Supplementary Material. The bootstrapped 95%-CIs of |α_12_|, shown also in Figure 2, are located clearly off an absolute difference of 0.0 (dotted line in the left panel of Figure 2) which would indicate both scaling exponents for short (below 1.5 h) and long (beyond 2 h) time ranges to be the same on average, and hence the presence of fractal scaling over the whole time range. Thus, DFA indicates a significant break-down of fractal scaling for the given sample. We obtained an analogous result in our analysis of CDDs. In particular, sample means of LLR are significantly larger than 0.0, again for all considered time resolutions, see Figure 2 (middle panel), i.e., lognormal distributions provide better fits to the CDDs than power law distributions (which would indicate fractal scaling). For the GOF ratios, a ratio of 1 indicates equal goodness of fit for both the lognormal and the power law distributions. Inspection of corresponding 95%-CIs of sample means yields that GOF ratios are significantly larger than 1 (right panel in Figure 2) indicating again a significant deviation from fractal scaling. Numeric values for sample means of LLR and GOF ratios and their 95%-CIs for all considered time resolutions are provided in Supplementary Table 5.

These results appear robust also against eventual effects of gender, mobility of the patients, and events such as falls and/or physical restraining. In particular, we decomposed the entire sample into samples for male and female patients, for autonomously mobile and (at least partially) immobile patients, for participants who never fell and fell at least once over their stays in hospital, and for participants who were never subject to physical restraining and those who were subject to physical restraining at least once during their stays in hospital. In all cases the respective means of all three considered quantities (|α_12_|, LLR, GOF) indicate significant deviations from fractal scaling. As mentioned in section “Subjects and Wrist Actigraphy,” three participants showed day-night-reversal. Excluding these participants from our analyses also resulted only in marginally different numbers. Our results for the subsamples concerning gender, mobility, falls, physical restraining and for the exclusion of participants with day-night-reversal are provided in Supplementary Tables 6–14.

However, we note substantially larger fluctuations with respect to time resolution for LLR and GOF ratios than for |α_12_|. This is a consequence of the considerably larger fluctuations with respect to time resolution of those quantities at the individual per-patient level. In particular, the scaling exponents as well as their (signed and unsigned) differences resulting from DFA appear to be converged within 0.02, 0.048, 0.041, and 0.041 for α_*1*_, α_*2*_, α_*12*_, and |α_12_| for time resolutions of at least CP30s. Here, we are taking the maximal span of ranges of those quantities for CP30s, CP15s, CP10s, and CP5s with respect to all patients as the most conservative measure of their convergence with respect to time resolution. Skipping the lowest time resolution (CPM) results from noting that for this resolution values can still be rather different from values at the other resolutions whereas for the latter they appear to be converged within the limits given above. We note that these measures of convergence may serve also as measures of accuracy of the DFA results with respect to time resolution. This could be extended also to the individual level in which case accuracies with respect to time resolution are mostly well below the above given most conservative estimates. The low values and hence the low fluctuations of individual DFA results underpin the robustness of the DFA results concerning time resolution. In contrast, analysis of CDDs results in highly fluctuating individual values for LLR and GOF ratios. Both quantities do not appear to yield any convergent behavior with increasing time resolution, i.e., individual values may change as much by decreasing time resolution from CPM to CP30s as for decreasing it from CP30s to CP15s or CP15s to CP10s, etc. Moreover, the mean spans of ranges for all considered time resolutions yield 2.22 (SD = 1.26; min = 0.59; max = 6.26) and 0.86 (SD = 0.49; min = 0.22; max = 2.38) for LLR and GOF ratios, respectively, hence yielding values of the same order of magnitude as the sample means themselves. Nevertheless, deviations from fractal scaling in case of CDDs are also clearly observable on an individual level of assessment by analyzing absolute frequencies of significantly better fits obtained for lognormal distributions than obtained for power law distributions to the individual data as described in section “Statistical Analyses.” This results in 22 (61%), 27 (75%), 29 (81%), 26 (72%), and 32 (89%) data sets out of 36 in total which are significantly better described by a lognormal than a power law distribution for the time resolutions of CPM, CP30s, CP15s, CP10s, and CP5s, respectively. For the remaining data sets none of the two distributions delivers a statistically significantly better fit to the data. Moreover, a power law distribution provides a significantly better fit to the data than a lognormal distribution in none of all cases for all considered time resolutions. Assuming that the functional form of the “true” CDD corresponding to the specific participant becomes captured the better the higher the time resolution, this represents rather strong evidence that a lognormal distribution provides overall a substantially better fit to the data than does a power law distribution.

Altogether, both kinds of analyses result in substantial deviations from fractal scaling both at an individual and at a sample level of assessment. Whereas results obtained with DFA are highly robust with respect to the used time resolution, individual values of the quantities obtained via analysis of CDDs are highly sensitive to time resolution, although the primary outcome of indicating a break-down of fractal scaling is not affected.

Finally, we report the mean values (with standard errors) of the scaling exponents obtained in the case of DFA for the two distinguished time regimes. For α_*1*_ we obtain 0.99 ± 0.01, 0.95 ± 0.01, and three times 0.94 ± 0.01 for the time resolutions of CPM, CP30s, CP15s, CP10s, and CP5s, respectively. For α_*2*_ we obtain 0.90 ± 0.02 for all considered time resolutions. Since the information contained in the magnitudes of the scaling exponents obtained in the case of analysis of CDDs could be highly misleading, see section “Discussion,” we refrain from reporting and interpreting them here. Further descriptive statistics also concerning the other quantities assessed in this work can be found in Supplementary Tables 2,  4.

### Assessment of Associations Between Determined Parameters

We report first the pair-wise correlation coefficients obtained for |α_12_|, LLR and GOF ratios for the five considered time resolutions, i.e., CPM, CP30s, CP15s, CP10s, and CP5s. The results are summarized in Tables 2–4 together with bootstrapped 95%-CIs for the computed correlation coefficients (below diagonal). For |α_12_| we robustly find high correlations with 95% confidence above at least 0.83 (lower bound of the 95%-CIs) which increase further to above 0.98 if the lowest time resolution of CPM is disregarded, see Table 2. This is in line with the already noted convergence of the obtained DFA parameters for time resolutions of at least CP30s, see section “Assessment of Deviations From Fractal Characteristics.” Moreover, this finding holds also for the individual scaling exponents α_*1*_ and α_*2*_ as well as their signed difference α_*12*_ for which correlations for time resolutions of at least CP30s are robustly above 0.99, see Supplementary Tables 15–17. Due to this result, we restrict our report of further associations between the quantities obtained via DFA and other parameters to a time resolution of CP15s for the remainder of this work without expecting any loss of generality.

**TABLE 2:** Pair-wise correlation coefficients (above diagonal) and bootstrapped 95%-CIs (below diagonal) for |α_12_| obtained via DFA of all participants’ activity data using the five considered time resolutions CPM, CP30s, CP15s, CP10s, and CP5s.

	**CPM**	**CP30s**	**CP15s**	**CP10s**	**CP5s**
CPM	1	0.928	0.902	0.897	0.897
CP30s	[0.876, 0.961]	1	0.991	0.989	0.991
CP15s	[0.839, 0.943]	[0.983, 0.996]	1	0.995	0.996
CP10s	[0.833, 0.940]	[0.980, 0.995]	[0.988, 0.999]	1	0.998
CP5s	[0.834, 0.939]	[0.984, 0.996]	[0.990, 0.999]	[0.997, 0.999]	1

In section “Assessment of Deviations From Fractal Characteristics,” we found a substantial dependence on the used time resolution concerning the LLR and GOF ratios obtained via analysis of CDDs on the used time resolution. This is also reflected in the pair-wise correlations, see Tables 3, 4. The highly fluctuating correlation coefficients are further evidence that interpretation of the assessed quantities must be exercised with caution. Their magnitude does probably not yield a reliable indicator of the amount of the individual deviation from fractal scaling. The sign of LLR, in especially being significantly larger than 0.0, or a GOF ratio significantly larger than one, can still serve as reliable indicators of the presence of a significant deviation from fractal scaling, though. The high degree of fluctuation in the magnitudes of these quantities for all considered time resolutions is reflected also in fluctuating correlations between corresponding LLR and the GOF ratios, see Table 5. The correlation coefficients can be considered rather low or at most moderate given that these measures represent different approaches to quantify the same property, i.e., the magnitude of the deviation of the CDD from a power law. Furthermore, both measures do not yield any significant correlation with the unsigned difference between the scaling exponents obtained via DFA, i.e., |α_12_|, see also Table 5. Due to these findings, we refrain from interpreting any correlations between those two quantities and other parameters, but we include the resulting pair-wise correlation coefficients in Supplementary Tables 18, 19 in order to tentatively show that the sensitivity of LLR and the GOF ratio on time resolution is associated also with corresponding fluctuations in those coefficients. Some methodological implications of these findings are discussed in more detail in section “Discussion.”

**TABLE 3:** Pair-wise correlation coefficients (above diagonal) and bootstrapped 95%-CIs (below diagonal) for LLR obtained via analysis of the CDDs of all participants using the five considered time resolutions CPM, CP30s, CP15s, CP10s, and CP5s.

	**CPM**	**CP30s**	**CP15s**	**CP10s**	**CP5s**
CPM	1	0.539	0.493	0.370	0.172
CP30s	[0.168, 0.732]	1	0.612	0.341	0.442
CP15s	[0.172, 0.719]	[0.366, 0.796]	1	0.709	0.639
CP10s	[0.044, 0.637]	[−0.032, 0.737]	[0.509, 0.866]	1	0.493
CP5s	[−0.155, 0.451]	[0.226, 0.660]	[0.414, 0.841]	[0.184, 0.795]	1

**TABLE 4:** Pair-wise correlation coefficients (above diagonal) and bootstrapped 95%-CIs (below diagonal) for the GOF ratios obtained via analysis of the CDDs of all participants using the five considered time resolutions CPM, CP30s, CP15s, CP10s, and CP5s.

	**CPM**	**CP30s**	**CP15s**	**CP10s**	**CP5s**
CPM	1	0.385	0.132	−0.053	−0.175
CP30s	[0.091; 0.655]	1	0.668	0.484	0.300
CP15s	[−0.238, 0.543]	[0.469, 0.819]	1	0.760	0.589
CP10s	[−0.357, 0.328]	[0.173, 0.806]	[0.598, 0.886]	1	0.689
CP5s	[−0.421, 0.113]	[−0.048, 0.631]	[0.299, 0.807]	[0.484, 0.830]	1

**TABLE 5:** Correlation coefficients and bootstrapped 95%-CIs for associations between LLR and GOF, LLR and |α_12_|, and GOF and |α_12_| for all considered time resolutions.

	**CPM**	**CP30s**	**CP15s**	**CP10s**	**CP5s**
*r*(LLR,GOF)	0.603	0.309	0.624	0.484	0.472
	[0349, 0.760]	[−0.153, 0.583]	[0.404, 0.773]	[0.190, 0.690]	[0.164, 0.691]
*r*(LLR,|α_12_|)	0.151	0.074	0.187	0.158	0.179
	[−0.109, 0.433]	[−0.260, 0.371]	[−0.160, 0.495]	[−0.200, 0.444]	[−0.137, 0.431]
*r*(GOF,|α_12_|)	0.011	0.170	0.158	0.141	0.063
	[−0.314, 0.303]	[−0.263, 0.462]	[−0.187, 0.469]	[−0.253, 0.450]	[−0.277, 0.371]

In Tables 6, 7, we supply pair-wise correlation coefficients together with their bootstrapped 95%-CIs for correlations between the DFA parameters α_*1*_, α_*2*_, α_*12*_, |α_12_| (obtained using a time resolution of CP15s) and the variables IS, IV, RA, M10, the age and the MMSE scores of the study participants. Note that MMSE scores were available only for 31 out of 36 participants. Besides the mathematically founded associations between α_*1*_, α_*2*_, and α_*12*_, i.e., α_*12*_ increases for increasing α_*1*_ as well as for decreasing α_*2*_ (recognizing, however, the dominant contribution of α_*2*_), we note that both α_*1*_ and α_*2*_ increase with M10, i.e., an increasing overall level of activity, *r*(α_*1*_, M10) = 0.490 with 95%-CI [0.201, 0.743], *r*(α_*2*_, M10) = 0.526 with 95%-CI [0.222, 0.707], i.e., the more active participants are associated with more regularity in their activity fluctuations at both short (below 1.5 h) and long (beyond 2 h) time scales. Based on their CIs, we note further that especially the scaling exponents α_*1*_ at short timescales and α_*2*_ at long time scales as well as all other pairs of quantities do not yield any significant (linear) associations between them. Moreover, we find a significant increase of the MMSE score with increasing α_*1*_, *r* = 0.415 with 95%-CI [0.060, 0.714], as well as with decreasing α_*2*_, *r* = −0.328 with 95%-CI [−0.549, −0.073]. Consequently, there is a significant even more pronounced association also for the signed difference α_*12*_ and the MMSE score, *r* = 0.494 with 95%-CI [0.227, 0.695], which is lost by omitting the sign (see the results for |α_12_| in Table 7).

**TABLE 6:** Pair-wise correlation coefficients (above diagonal) and bootstrapped 95%-CIs (below diagonal) for the DFA parameters α_1_, α_2_, α_12_, |α_12_| (obtained using the time resolution CP15s).

	**α_1_**	**α_2_**	**α_12_**	**|α_12_|**
α_1_	1	0.008	0.474	0.271
α_2_	[−0.306, 0.272]	1	−0.876	−0.172
α_12_	[0.173, 0.740]	[−0.930, −0.797]	1	0.282
|α_12_|	[−0.039, 0.563]	[−0.647, 0.257]	[−0.128, 0.743]	1

**TABLE 7:** Pair-wise correlation coefficients (above diagonal) and bootstrapped 95%-CIs (below diagonal) between the DFA parameters α_1_, α_2_, α_12_, |α_12_| obtained using the time resolution CP15s and the variables IS, IV, RA, M10, the age and the MMSE scores of all study participants.

	**IS**	**IV**	**RA**	**M10**	**Age**	**MMSE^a^**
α_*1*_	0.317	0.110	0.172	0.490	0.017	0.415
	[0.018, 0.586]	[−0.240, 0.438]	[−0.121, 0.442]	[0.205, 0.743]	[−0.323, 0.360]	[0.060, 0.714]
α_*2*_	0.266	−0.876	0.092	0.526	−0.140	−0.328
	[−0.053, 0.538]	[−0.926, −0.818]	[−0.174, 0.339]	[0.222, 0.707]	[−0.372, 0.058]	[−0.549, −0.073]
α_*12*_	−0.081	0.825	0.003	−0.226	0.132	0.494
	[−0.460, 0.311]	[0.708, 0.900]	[−0.270, 0.286]	[−0.542, 0.215]	[−0.143, 0.410]	[0.227, 0.695]
|α_12_|	0.301	0.398	0.250	0.165	−0.144	0.217
	[0.010, 0.562]	[0.020, 0.729]	[−0.014, 0.476]	[−0.163, 0.459]	[−0.400, 0.119]	[−0.152, 0.566]

*^*a*^MMSE scores were available only for 31 out of 36 participants.*

We further find slight associations between the interdaily stability of activity, IS, and the scaling exponent at short time scales (below 1.5 h), α_*1*_, *r* = 0.371 with 95%-CI [0.018, 0.586] and also between IS and the unsigned difference of scaling exponents, |α_12_|, *r* = 0.301 with 95%-CI [0.010, 0.562]. The parameter IS is also strongly associated with the rhythm amplitude, RA, *r* = 0.745 with 95%-CI [0.594, 0.874] which quantifies the difference in activity between the ten most active and five least active hours and it is thus methodologically also associated with the parameter M10. Indeed, all three parameters, IS, RA, and M10 are definitely intertwined as can be seen also from inspection of Table 8 which, for the sake of completeness, provides the pair-wise correlation coefficients together with their bootstrapped 95%-CIs obtained between the circadian parameters IS, IV, RA, M10, the age, and the MMSE scores of all study participants. All three parameters also yield at least a tendency to decrease with increasing age, see Table 8. Furthermore, we find a strong association between the scaling exponent at long time scales (beyond 2 h), α_*2*_, and the intradaily variability, IV, *r* = −0.876 with 95%-CI [−0.926, −0.818], i.e., IV decreases for increasing α_*2*_. This can be understood by reflecting the methodological foundations of both quantities. The scaling exponent α_*2*_ quantifies the regularity of activity fluctuations at time scales beyond 2 h. On the other hand, IV quantifies how strongly the actigraphic signal varies on a daily basis by accumulating activities into hourly values (Blume et al., 2016). A more regular actigraphic signal is characterized by a higher probability of low/high activities being followed by low/high activities compared to a more irregular signal for which such associations are less pronounced. Assessing this characteristic on time scales of the order of hours, IV will increase the more irregular an actigraphic signal is, and by construction, the opposite is true for α_*2*_. Hence, the strong association between those two quantities is merely based on common method variance and thus, unsurprising. The further significant associations between the signed and unsigned differences, α_*12*_ and |α_12_|, and IV, see Table 7, are then a direct consequence of this methodologically founded association between α_*2*_ and IV. Consequently, also IV and the MMSE score are associated with each other, *r* = 0.411 with 95%-CI [0.108, 0.663], see Table 8.

**TABLE 8:** Pair-wise correlation coefficients (above diagonal) and bootstrapped 95%-CIs (below diagonal) for the circadian parameters IS, IV, RA, M10, the age (in years), and the MMSE scores of the participants.

	**IS**	**IV**	**RA**	**M10**	**Age**	**MMSE^a^**
IS	1	−0.025	0.745	0.654	−0.326	0.103
IV	[−0.325, 0.311]	1	0.019	−0.350	0.127	0.411
RA	[0.546, 0.893]	[−0.265, 0.344]	1	0.357	−0.412	0.000
M10	[0.464, 0.800]	[−0.610, 0.040]	[0.065, 0.603]	1	−0.242	0.142
Age	[−0.631, 0.022]	[−0.084, 0.308]	[−0.642, −0.119]	[−0.548, 0.049]	1	−0.058
MMSE	[−0.276, 0.494]	[0.108, 0.663]	[−0.480, 0.392]	[−0.214, 0.582]	[−0.473, 0.275]	1

*^*a*^MMSE scores were available only for 31 out of 36 participants.*

The reported associations between the assessed variables are also not affected substantially by exclusion of the three participants with day-night-reversal. The corresponding data are provided in Supplementary Tables 20–22.

## Discussion

We found evidence that both locomotor activity signal characteristics considered in this work [i.e., activity fluctuations and complementary cumulative distributions of low-activity durations (CDDs)] deviate from fractal scaling over the entire time range from about 1 min to about 10 h for our sample of 36 geriatric in-patients with AD. In the case of activity fluctuations assessed via DFA, our results indicate rather the existence of two distinct scaling regimes. Our measure of deviance from fractal scaling over the entire time range in that case, i.e., the absolute value of the difference of scaling exponents at short (below 1.5 h) and long (beyond 2 h) time scales |α_12_|, turned out to be highly robust with respect to the chosen time resolution for obtaining the analyzed activity signal. In particular, for the assessed time resolutions below 1 min, i.e., accumulation of activity counts per 30, 15, 10, and 5 s, said measures, represented by numbers between 0.5 and 1.5, have been converged within at least 0.02 for our sample. This can serve as an estimate of the accuracy with which these measures are determined by DFA with respect to time resolution of the analyzed signal. Similar accuracies resulted for the individual scaling exponents as well as the signed differences of them obtained via DFA. We can hence add this finding to the already established robustness of the quantities obtained with this method against external schedules, individual average activity levels and circadian phase (Hu et al., 2004).

While we could thus advise to use time resolutions of or below 30 s in future work utilizing DFA for the analysis of human locomotor activity, we would rather emphasize on adding an assessment of the variation of resulting scaling exponents with respect to time resolution to the analysis protocol in order to infer about the accuracy of the obtained results with respect to this technical choice. Nowadays, many actigraphs allow access to the raw, recorded accelerations (John and Freedson, 2012) and hence, straightforward computation of appropriate activity signals for further analysis using various time resolutions for the latter. Since the resulting accuracy with respect to time resolution can, in principle, depend on the specific sample or other study parameters, further independent assessments of this accuracy would be informative concerning the generalizability of our finding while requiring manageable additional effort.

In contrast, the measures assessing the deviation from fractal scaling upon analyses of CDDs depended strongly on the time resolution of the underlying signal. While the presence of a substantial deviation from power law like CDDs *per se*, both at sample and individual level of assessment, was a common feature for all considered time resolutions, the individual magnitudes of the assessed measures for it turned out to be highly varying for different time resolutions. We hence refrained from interpreting the latter, noting merely that there is clear evidence for a deviation from fractal scaling also in the case of analysis of CDDs.

For the analyses of CDDs, the assessment of fractal characteristics was based on the comparison of how well a power law or a lognormal distribution could describe the empirical data of an individual participant. One reason for the sensitivity of the resulting measures, represented by two kinds of ratios of relative goodness-of-fit parameters, could then be that neither the power law nor the lognormal distribution provide optimal fits to the empirical data. This is indicated already at the level of bare inspection of graphs of the various empirical CDDs which we provide also in the Supplementary Material. Indeed, we found that those graphs can be categorized into three groups: (i) four instances in which both the power law and the lognormal distribution appear to provide good fits to the data, (ii) three instances in which both distributions appear to provide only poor fits to the data, and (iii) 29 instances in which the lognormal distribution provides considerably better fits to the data than power law distributions. However, in most of these cases (20 of 29 instances) lognormal distributions appear to provide better fits than power laws to the CDDs beyond 10–20 min, whereas the best fitting power laws typically yield considerably shorter minimum durations (i.e., $d_{\text{min}}^{\text{PL}}$ below 10–20 min). Generally, it could be the case that the specific functional form of the analyzed CDDs eventually corresponds rather to a composition of, e.g., a power law like function at shorter time scales and the cumulative distribution function of a lognormal distribution at longer time scales. The actual functional form of the CDDs may then contain more (also psychologically) relevant information than can be resolved by our analysis. Comparing the CDDs of patients diagnosed with psychotic or bipolar disorder yielded the result that different psychopathological states may be related to different, specific functional forms of CDDs which were, in the considered cases, not represented by simple power laws (Chapman et al., 2017). We hence conclude that such an analysis may well provide an interesting route for future research also in the present context of dementia.

The finding that a power law does not provide a good fit for the assessed data for most of the participants has consequences also for quantities derived from the empirical CDDs as well as interpretations based on them. Nakamura et al. (2007, 2016) proposed a model for the temporal coordination of locomotor activity using the stochastic priority list model by Barabási (2005). The authors assume that spontaneous motoric activity is triggered by responses to internal or external demands or stimuli such as appetite, emotion, etc. (Nakamura et al., 2016). If stimuli or demands are probabilistically chosen in direct proportion to their biological importance, then the CDDs, resulting from the model, would follow a power law with a scaling exponent γ = 1 (Nakamura et al., 2016). Preferential selection of stimuli or demands with higher priorities (relative to the selection of lower-priority stimuli or demands) would result in scaling exponents lower than one. This would give rise to more frequent longer low-activity periods and a more intermittent sequence of onsets of activity bursts as observed in major depression (Nakamura et al., 2007), bipolar disorder during the depression phase (Nakamura et al., 2013a) and schizophrenia (Sano et al., 2012). The opposite, i.e., preferential selection of lower-priority stimuli or demands would instead result in scaling exponents larger than 1 and were observed in bipolar disorder during a manic episode (Nakamura et al., 2013a). The model could thus provide a promising link between the functional form of the empirically obtainable CDD and an eventual underlying psychopathological state. The scaling exponents were hence discussed as eventual non-invasive biomarkers for the assessed pathological states (Kim et al., 2016; Nakamura et al., 2016).

However, in the present context it is important to note that the proposed model suggests the empirical CDDs to follow a power law distribution (at least over time scales ranging from a few minutes to several hours) in all of the discussed cases. Hence, empirical CDDs deviating substantially from scale invariance are simply not in the scope of the model and therefore should not be interpreted on its basis. This is especially important since technically, a power law distribution can be fitted to CDDs in any case regardless if they adhere in principle to a power law distribution or not. Then, however, the resulting scaling exponents are merely a consequence of the actual functional form of the CDDs and parameters and adjustments with respect to the fitting procedure, but certainly not of relations derived via a model applicable only if the empirical CDDs yield the functional form of a power law. This is reflected in the present study also by noting that the dominant portion of the variance in the computed scaling exponents can be explained by the choice of the parameter *d*_*min*_ determined during the fitting procedure (see section “Materials and Methods”) and the maximal duration of low-activity periods in the respective data, see also Supplementary Tables 2–4. Using these two variables as predictors results indeed in highly significant regression equations explaining 58–75% of the variance of the scaling exponents γ for all considered time resolutions, see Supplementary Table 3. Obviously, this is at cross with the notion of (time) scale invariance, but is an expectable result if the assessed CDDs are simply not well described by power laws in the considered time range.

This finding calls also for additional conceptual groundwork. The model proposed by Nakamura et al. (2007, 2016) provides a link between the temporal coordination of general motoric activity and cognitive decision-making and prioritization processes in response to internal and external influences. Nevertheless, the model cannot account for CDDs substantially deviating from the functional form of a power law. In line with our findings, it has been noted earlier (Chapman et al., 2017) that the stochastic models which may best describe motoric behavior are more complex than previously thought. Chapman et al. (2017) assessed CDDs from patients with psychotic symptoms or bipolar disorder and found that they were well described by truncated power laws as well as sums of exponentials and truncated power laws in contrast to power laws as such. Furthermore, patients with chronic pain yield CDDs which can be described better by the distribution function of a lognormal distribution than of a power law in 60% of the cases (Paraschiv-Ionescu et al., 2013). Working hence on conceptual extensions of the model proposed by Nakamura et al. (2007, 2016) may eventually lead to an increased understanding of the interaction of different functional, neural systems, which are probably involved in generating the complex dynamics reflected in the presence of long-tailed statistics in locomotor activity data (Roberts et al., 2015). Such an approach would require to focus especially on how memory and its decline may affect or can be incorporated in the mathematical formulation of the model. In turn, a better fundamental understanding could then foster the utilization of adequate analyses of locomotor activity data for providing information about diagnostically relevant neural processes via completely non-invasive actigraphy. However, toward this ambitious, long-term goal a variety of potential complications and difficulties need to be considered and scrutinized, which shall be outlined below with regard also to the various limitations of the present study.

While our results provide evidence of substantial deviations from scale invariance in the assessed locomotor activity data, these deviations cannot be attributed simply to the prevalence of dementia in our sample. Nevertheless, earlier findings indicate that at least in the case of the DFA results, a significant portion of the noted effect is probably due to the cognitive decline associated with dementia. Hu et al. (2009) found that scaling exponents obtained via DFA at time scales in a range of 1.5-8 h decline with age and are, independently of age, even further reduced in patients with AD. This was further associated with attenuated functionality of the suprachiasmatic nucleus (SCN) due to anatomical and physiological changes with aging and AD, suggesting the respective scaling exponents as non-invasive biomarkers for SCN function (Hu et al., 2009). Interestingly, in our correlational analyses, we find a strong, negative association between the scaling exponent α_*2*_ and the circadian parameter IV (*r* = −0.876, 95%-CI [−0.926, −0.818]). The higher irregularity in the activity signal reflected by these quantities is likely to be related with SCN function and sleep disturbances (Scherder et al., 1999; Huang et al., 2002; Hatfield et al., 2004; Musiek et al., 2018). However, we obtain also the somewhat controversial results of a negative association between the scaling exponents α_*2*_ and the MMSE scores of our present participants (*r* = −0.328, 95%-CI [−0.549, −0.073]), and in accordance with the foregoing discussion, also a positive association between the circadian parameter IV and the MMSE scores. To our best knowledge such a correlation between activity (ir-)regularity at longer time scales and MMSE scores has not been reported so far, and at this moment, we lack an explanation for this result.

The relevance of fractal characteristics at longer time scales beyond 1.5-2 h puts emphasis also on the possibility to decouple different time scales within DFA. We particularly obtain no significant correlation between α_*1*_ and IV in line with the expectation that changes in the SCN affect activity regulation only at longer time scales (Hu et al., 2007, 2009). Moreover, Hu et al. (2013) used especially the (signed) difference between scaling exponents at short and long time scales to quantify the degree of disturbed fractal regulation. They found that a more pronounced disruption, i.e., a larger difference between the two scaling exponents for short and long time scales (α_*1*_ and α_*2*_ in the present work, respectively), is associated with more amyloid plaques in the occipital cortex. The finding suggests that patients with more severe AD yield also proportionally enhanced deviations from scale-invariant activity fluctuations (Hu et al., 2013). Furthermore, this difference measure served as the most significant predicting factor explaining most of both the variation of vasopressin and of neurotensin in the SCN. Therefore, this measure led to a vast improvement in predicting changes in these circadian neurotransmitters in comparison to more traditional markers such as the circadian parameter IV (Hu et al., 2013).

Moreover, the finding that AD appears associated with the break-down of fractal scaling into the discussed two distinct time regimes (Hu et al., 2009, 2013), which is further corroborated by our present results, suggests also an eventual utilization of multifractal approaches (França et al., 2019) for the characterization of neurodegenerative diseases. Multifractal analysis could provide an indirect measure to distinguish patients suffering from fibromyalgia from healthy subjects, whereas a monofractal approach yielded no significant difference between the two samples (França et al., 2019). Due to the existence of distinct scaling regimes in the case of AD patients in contrast to healthy subjects, it can be expected that such a multifractal approach allowing the derivation of multifractal spectra can reveal a more finely resolved picture of the associated difference in fractal regulation between AD patients and healthy subjects. Furthermore, between both AD (Witting et al., 1990; van Someren et al., 1996) and fibromyalgia (Wolfe et al., 1990) exist associations with disturbances in the sleep-wake cycle or rest-activity rhythm, which provides at least a further indication for a useful application of multifractal methods also in the case of AD. Future studies aiming at scrutinizing the capabilities of multifractal methods as eventual diagnostic tools in the framework of neurodegenerative diseases appear hence as one promising route to follow. In this context, we would like to note also that the existence of well-distinct scaling regimes is hardly an exclusive phenomenon to general locomotor activity in AD. For instance, two distinct scaling regimes have repeatedly been found also in the analysis of center of pressure trajectories of subjects during quiet standing (Collins and De Luca, 1994; Blázquez et al., 2009, 2012). This finding was associated with the inclusion of both open-loop and closed-loop control processes in the human postural control system (Collins and De Luca, 1994), corroborating hence the view of the analysis of fractal signal characteristics as a feasible tool to (indirectly) shed some light on underlying neuro-physiological mechanisms.

In 2016, Hu et al. (2016) reported that especially scaling exponents at the shorter time scales (α_*1*_) are reduced as AD progresses and may hence be utilized for long-term monitoring of dementia progression. Similarly to the present study, they found significant positive associations between the scaling exponents α_*1*_ and the MMSE scores of their patients at baseline (*r* = 0.25). In a recent study, Li et al. (2018) reported that α_*1*_ may even be useful for predicting the risk of AD and the speed of cognitive decline, independently from other risk factors like physical activity, sleep fragmentation and the stability of daily activity rhythms. Taken together, the discussed findings support the notion that the fractal characteristics of locomotor activity data contain valuable information concerning changes in neural function and cognitive decline which also appear not to be entirely masked by the typical complication of a highly heterogeneous sample in the case of AD.

Completing our discussion of the correlational analyses performed by us, we note positive associations between our measure for average overall activity and the magnitudes of both scaling exponents α_*1*_ and α_*2*_. Given that the overall means of both scaling exponents are smaller than 1, this accords to the expectation that both higher average motoric activity as well as scaling exponents closer to 1 tend to be signs of greater health. In particular, we obtained mean values of 0.94–0.99 ± 0.01 for α_*1*_ (depending somewhat on the considered time resolution) and 0.90 ± 0.02 (irrespective of time resolution) for α_*2*_. Although the focus of our study is on the assessment of the deviation from fractal scaling (or the break-down into two distinct scaling regimes in the case of DFA), it appears noteworthy to discuss also the magnitudes of these scaling exponents in comparison with earlier investigations. For short time ranges (below about 1.5 h) scaling exponents of 1.00 ± 0.02 for 13 elderly (mean age: 68.5 years, SD = 6.1 years) early stage AD subjects, 0.94 ± 0.03 for 14 very old (mean age: 83.9 years, SD = 6.7 years) late-stage AD subjects (Hu et al., 2009), 1.13 ± 0.03 for 20 elderly (62–80 years) subjects (Hu et al., 2013) with various forms of dementia, but mostly AD, and 0.97 ± 0.01 for 165 subjects (70–96 years) with mostly AD but also other forms of dementia (Hu et al., 2016) have been reported earlier. Although there appears some variance across the different samples, our result appears reasonable in comparison with the mentioned ones and also with the (apparently normal) distribution of the scaling exponent at small time scales reported by Li et al. (2018) for a large sample of 1097 non-AD subjects (mean age: 81 years, SD = 7.4 years) yielding a median of 0.92 (SD = 0.06) and a range of exponents from about 0.6 to about 1.15. Concerning the scaling exponent for longer time scales (above about 2 h), mean values of 0.80 ± 0.03 for 13 elderly (mean age: 68.5 years, SD = 6.1 years) early stage AD subjects, 0.69 ± 0.03 for 14 very old (mean age: 83.9 years, SD = 6.7 years) late-stage AD subjects (Hu et al., 2009), 0.88 (with a standard error of 0.05 for the difference between the two distinct scaling exponents) for 15 elderly (62–80 years) subjects (Hu et al., 2013) with various forms of dementia, but mostly AD, and 0.72 ± 0.01 for 165 subjects (70–96 years) with mostly AD but also other forms of dementia (Hu et al., 2016) have been reported. In contrast, 13 elderly (mean age: 68.6 years, SD = 6.1 years) and 12 very old (mean age: 80.8 years, SD = 8.6 years) control subjects yielded exponents of 0.83 ± 0.03 and 0.79 ± 0.04, respectively, hence showing that also age appears to affect fractal regulation at longer time scales (Hu et al., 2009). Furthermore, 13 young control subjects (mean age: 25.5 years, SD = 6.1 years) yielded a mean scaling exponent 0.91 ± 0.02 over the entire considered time range from minutes up to 8 h (Hu et al., 2009) in good agreement with an earlier study (Hu et al., 2004) yielding a scaling exponent of about 0.9 for 16 young subjects (19–44 years). In view of these results, the obtained mean value of α_*2*_ = 0.90 ± 0.02 for our sample appears at the higher end of the range of reported values for samples with dementia and intriguingly close to the value reported for young control subjects. However, while all the discussed studies have in common their focus on dementia, a single factor shared between our study and the one of Hu et al. (2013), reporting a similar, large scaling exponent of 0.88 for longer time scales, is that they both consider in-patients. In contrast, the above mentioned studies yielding considerably lower scaling exponents at longer time scales explored daytime activity patterns of subjects maintaining their habitual sleep-wake schedules (Hu et al., 2013) or considered locomotor activity data of assisted care facility residents (Hu et al., 2016). Although we cannot rule out cultural effects (the discussed earlier investigations were conducted in the United States and Netherlands) or effects stemming from different sample heterogeneities with respect to comorbidities or medication, we think that the largely standardized activity-protocols usually followed in hospital treatment may have an impact also at the fractal regulation of locomotor activity, especially at longer time scales ranging over several hours. This appears at least in line with the close associations found between the respective scaling exponent and circadian parameters discussed above. This expectation could be basically tested by a direct comparison of the fractal regulation of locomotor activity in AD in- and out-patients (ideally, in addition to a sample of age-matched control subjects).

The clinical setting of our study is naturally associated with a number of limitations. In particular, our participants yielded a large amount of comorbidities, some of which may also be related to symptoms of (chronic) pain and, obviously, high heterogeneity in administered medication. Chronic pain cannot be excluded from affecting the functional form of CDDs (Paraschiv-Ionescu et al., 2013) and future studies will be required to scrutinize likely interactions with effects stemming from dementia. Dedicated large-scale research is likely to be needed to resolve the various conceivable consequences of a probably highly nested network of relevant variables (if this is possible at all). Moreover, we could not (or just to a very limited extent) investigate the influence of demographic characteristics (age, social affiliations, etc.), different medical interventions (ranging from exercise therapy to physical restraining), or other variants of neurodegenerative diseases (vascular dementia, frontotemporal dementia, Lewy body dementia, Parkinson’s disease) on the assessed fractal characteristics. Although we obtained clear deviations from fractal scaling for both autonomously mobile and at least partially immobile patients, subtler effects on the fractal characteristics of activity signals stemming from the patients’ mobility as well as dynamic effects associated with comparatively rare events such as falls cannot be excluded. The same holds for our comparison of patient samples experiencing and not experiencing physical restraining. Finally, it has been shown that the overall scaling exponents obtained via DFA are independent of external schedules, reactions to the environment, average activity levels, the phase of the circadian pacemaker and the placement of the actigraph at either the dominant or non-dominant wrist (Hu et al., 2004). Reproduction of these findings for a sample of geriatric patients with dementia like in the present context is, to our best knowledge, yet lacking. However, this would require a decomposition of the entire time scale into separate time ranges able to describe an eventual deviation from fractal scaling.

More technical issues, which nevertheless may need further elaboration before any clinical, diagnostic application of the considered analyses, are (a) the amount of required activity data for adequate analyses, (b) effects concerning the placement of the actigraph, (c) effects of differently defined thresholds for decomposing the overall activity signal versus threshold-free analyses, (d) the eventual necessity to exclude certain data (like day and/or night activity), and – as discussed already above – (e) the statistical models taken into account when analyzing the determined CDDs. Concerning (a), more than 9 h of recorded activity data should be enough to reliably estimate DFA scaling exponents for time scales below 1.5 h (Hu et al., 2001, 2004, 2009). Concerning analyses of CDDs, recordings of more than 7 days have been suggested (Sano et al., 2012). Moreover, DFA parameters were found to be stable across days in healthy, young subjects (Hu et al., 2004). If and to what extent these findings and suggestions may be transferable to geriatric in-patients with dementia remains yet an open issue. Concerning (b), we note that in their analyses of CDDs, Chapman et al. (2017) used hip-worn actigraphs and the elaboration of the impact of the placement of the actigraph on the outcome of different analyses represents an important question. This concerns also ideas to eventually utilize sensors already present in, e.g., mobile phones in contrast to the application of an additional, specific device, which is frequently not easily accepted by patients, especially when suffering from dementia. Concerning (c), we note that in the present study, we used the overall average activity to discriminate low-activity periods from elevated activity in the case of analyses of CDDs, a strategy which has been applied repeatedly in earlier investigations (Nakamura et al., 2007, 2008, 2013a,b, 2016; Sano et al., 2012; Chapman et al., 2017). Previously, it has been pointed out (Chapman et al., 2017) that this data-driven approach may be more sensitive to differences in activity patterns. However, alternative methods using externally validated thresholds based on categories of energy-expenditure might be more useful for determining, e.g., the time spent at activities of different intensities, but may also produce large discrepancies in outcomes. Threshold-free analyses could provide a further alternative (Chapman et al., 2017). Concerning (d), we note that exclusion of certain portions of the recorded activity data such as “sleep” or night data may appear desirable under certain circumstances, e.g., when the focus should be more on higher order, conscious processes. However, depending on the type of exclusion strategy, this can be typically associated with its very own pitfalls and shortcomings due to sleep latency, night time awakenings, day-night reversals, napping during the day or particularly restless sleeps among other factors.

## Conclusion

To summarize, we found significant deviations from fractal characteristics of locomotor activity regardless of the choice of the method, i.e., DFA or analysis of CDDs, for our sample of geriatric in-patients with dementia at both individual and sample levels of assessment. Further research is required to scrutinize whether and how much of this effect can be traced back to (a) the presence, severity and specific form of dementia, (b) comorbidities and their associations with pain, (c) effects of interventions including especially medication, and also how these possible causal factors may interact. However, taking together our results with the outcomes of earlier investigations (Hu et al., 2004, 2007, 2009, 2013, 2016; Li et al., 2018), we arrive at the conclusion that the fractal characteristics at least as quantified in terms of parameters obtained via DFA can be informative in neuropsychiatric contexts and that our study can add some credibility to the ecological validity of those parameters. Besides that, together with more conceptual groundwork, further investigations could clarify also general relations and interactions between the various functional, neural systems associated with motor preparation and coordination generating the complex dynamics apparent in everyday locomotor activity at the macroscale (Kelso, 1995, 2010; Van Orden et al., 2005).

Nevertheless, we also tried to emphasize that there exists a variety of open issues and complications which need further rigorous, scientific scrutiny. The possible benefits may, however, outweigh the required endeavors, if they would finally result in a set of non-invasive, methodologically robust biomarkers, eventually able to capture cognitive decline and the progression of dementia (Hu et al., 2016) and providing possibly also an early warning system regarding detrimental changes over the life-span and mortality risk (Raichlen et al., 2018) in a more general sense. The latter is especially important since concerning neurodegenerative diseases such as AD, interventions are typically more effective at an earlier stage (Caselli and Reiman, 2013; Cummings et al., 2016). This finally puts emphasis on the importance of steadily supporting and promoting humane living and working conditions also on a societal level, given the impact which aspects of everyday life such as physical exercise (Gu et al., 2015) or chronic shift work (Li et al., 2017) can have on fractal characteristics of locomotor activity in light of the latter’s intricate relations with human health.

## Data Availability Statement

The authors confirm that some access restrictions apply to the data underlying the findings. The data cannot be made publicly available because informed consent from study participants did not cover public deposition of data. Requests to access the datasets should be directed to JM (josef.marksteiner@tirol-kliniken.at) or MC (markus.canazei@bartenbach.com).

## Ethics Statement

This study was in accordance with the Declaration of Helsinki and was approved by the ethics committee of the Medical University of Innsbruck, Austria, including all consent procedures described in the following. Written informed consent was obtained from all patients or legal representatives prior to participation. Written informed consent was given by the participants’ legal representatives if patients were unable to give informed consent due to mental incapacity. These patients had to have a legal representative before they were admitted to the study.

## Author Contributions

SH was involved in the following stages of the research process: conception, data processing, analysis and interpretation of the data, writing and composing the manuscript, and visualization of the results. MC, JM, WP, and EW were involved in conception and design, data collection, and in contributing to the writing of the manuscript. PS was involved in analysis and interpretation of the data and in contributing to the writing of the manuscript. AM was involved in analysis and interpretation of the data, in contributing to the writing of the manuscript, and in visualization of the results.

## Conflict of Interest

The authors declare that the research was conducted in the absence of any commercial or financial relationships that could be construed as a potential conflict of interest.
