# Supplementary material for: Assessment of Fractal Characteristics of Locomotor Activity of Geriatric In-Patients With Alzheimer’s Dementia
Source: Front Aging Neurosci. 2019 Oct 4;11:272. doi: 10.3389/fnagi.2019.00272 (PMC6787148; doi:10.3389/fnagi.2019.00272)
Supplement: Supplementary file 1 [file Data_Sheet_1.PDF]

Supplementary Material

Contents

1 Table of comorbidities 2

2 Descriptive statistics of DFA, CDD and circadian parameters 3

3 Dependence of measures of fractal characteristics on time resolution, gender, mobility and presence of day-night-reversal, falls and physical restraining 5

4 Correlation tables 8

5 Graphs of individual CDDs including PL and LN fits 11

5.1 Empirical CDDs according to Group 1.a 12

5.2 Empirical CDDs according to Group 1.b 15

5.3 Empirical CDDs according to Group 1.c 22

5.4 Empirical CDDs according to Group 2 26

5.5 Empirical CDDs according to Group 3 28

1 Table of comorbidities

In Table 1, those comorbidities reported for the 36 in-patients included in the present study, which yield absolute abundancies of four or higher in the whole sample, are listed in form of their ICD-10 codes and short verbal descriptions together with their absolute and relative abundancies in descending order of the latter. Those comorbidities which occurred more rarely, i.e. with absolute abundancies of three or less (below 8% in terms of relative abundancies) are just listed in terms of their respective ICD-10 codes (also to ensure the patients' anonymity). Note that concurrent mental and behavioral disorders other than dementia in Alzheimer disease (F00) or delirium superimposed on dementia (F05.1) have been exclusion criteria in the present study. We note also that the patients received a variety of medication which also changed in some cases considerably over their stays in hospital. On average [SD] they received 7.65 [4.60] (records missing for 8 patients) and 7.76 [2.19] (records missing for 4 patients) different pharmaceuticals at admission and at discharge, respectively.

Table 1: Table of comorbidities and their abundancies.

ICD-10 code	Short Description	Absolute abundance	Relative abundance (%)
I10	Essential (primary) hypertension	15	42
N39.0	Urinary tract infection	14	39
E53.8	Deficiency of other specified B group vitamins	8	22
E87.6	Hypokalemia	7	19
I25.1	Atherosclerotic heart disease of native coronary artery	6	17
G45.9	Transient cerebral ischemic attack	5	14
J44.9	Chronic obstructive pulmonary disease	4	11
E03.9	Hypothyroidism	4	11
N18.9	Chronic kidney disease	4	11
K59.0	Constipation	4	11
E87.0, H91.9, L89.9, T14.2, M48.0, J18.9, I48.0, Z86.7, C61, M47.9, E86, R29.6, T78.4, C18.9, I45.1, H40.1, K76.0, G40.9, M19.9, I50.9, I48.9, M17.9, E11.9, R55, G20, M81.9, I48.2, H40.9, H26.9, N17.9, R31, E05.9, B35.6, S72.0, G93.1, I44.3, Z96.6, M25.5, E21.3, Q61.3, K44.9, K21.0, R23.8, R60.1, K81.0, M13.1, C44.9, K64.9, J45.9, G44.2, S40.0, N39.3, C50.9, I51.7, B37.9, I70.0, M81.5, K76.9, I63.5, I71.2, J37.0, E78.0, L29.9, S80.0, S06.9, D86.9, N18.3, Z88.0, I62.0, I08.0, E04.0, I63.9, Z90.1, T14.1, I83.9, M17, G25.0, Z91.5, K25.9, I26.9, M75.4, T78.3, J02.9, T94.1, I70.9, T78.4, I34.0, B37.3, L23.0, M81.8, H91.1, M54.5, R32, N18.4, I50.0, I74.9, A09.9, L30.4, L30.9, I44.7, N40, N20.0, S72.1, S00.9, E56.9, D47.2, G60.9,		≤3	≤8

2 Descriptive statistics of DFA, CDD and circadian parameters

In this section, we supply descriptive statistics (means and standard deviations in parenthesis) for the various quantities analyzed in this study (i.e. the DFA parameters α_1, α_2, α_{12} and $|\alpha_{12}|$, the various quantities obtained via analysis of CDDs including LLRs based on minimum durations obtained for both power law as well as lognormal fits, number of data sets (n) which are significantly better fit using a lognormal distribution than a power-law, GOF ratios, said minimum durations and also maximum durations ($d_{\max}$) of low-activity intervals, as well as scaling exponents for the best fitting power law distributions, and finally the computed circadian parameters IS, IV, RA, L5, M10), for all considered time resolutions, i.e. counts-per-minute (CPM), counts-per-30-seconds (CP30s), counts-per-15-seconds (CP15s), counts-per-10-seconds (CP10s) and counts-per-5-seconds (CP5s). For a description of said quantities, see Section 2 of the main article. We include our results concerning the multiple linear regressions predicting the individually computed scaling exponents for the power laws fitted to the individual, empirical CDDs based on the minimum and maximum durations of low-activity intervals used in the fitting procedure, see Section 4 of the main article.

Table 2: Descriptive statistics for the various quantities analyzed for the 36 participants in this study.

Variable	CPM	CP30s	CP15s	CP10s	CP5s
α_1	0.986 (0.079)	0.948 (0.078)	0.941 (0.079)	0.939 (0.079)	0.939 (0.079)
α_2	0.904 (0.140)	0.903 (0.143)	0.902 (0.144)	0.904 (0.144)	0.903 (0.144)
α_{12}	0.082 (0.157)	0.045 (0.162)	0.039 (0.164)	0.035 (0.163)	0.036 (0.163)
$\|\alpha_{12}\|$	0.141 (0.105)	0.136 (0.097)	0.137 (0.094)	0.135 (0.094)	0.136 (0.094)
LLR (obtained using $\boldsymbol{d}_{\min}^{\mathrm{LN}}$)	1.930 (0.965)	2.278 (1.054)	2.681 (0.988)	2.468 (1.342)	3.251 (1.547)
LLR[a] (obtained using $\boldsymbol{d}_{\min}^{\mathrm{PL}}$)	-0.601 (0.669)	-0.894 (0.745)	-0.915 (0.802)	-0.734 (0.764)	-0.840 (1.008)
n	22 (61%)	27 (75%)	29 (81%)	26 (72%)	32 (89%)
GOF ratio	1.435 (0.486)	1.387 (0.400)	1.424 (0.450)	1.485 (0.610)	1.436 (0.539)
γ	1.350 (0.515)	1.275 (0.432)	1.214 (0.526)	1.332 (0.66)	1.244 (0.54)
$d_{\min}^{\mathrm{PL}}$ (min)	33.8 (31.8)	20.7 (22.8)	11.3 (15.2)	11.6 (13.7)	6.7 (9.1)
$d_{\min}^{\mathrm{LN}}$ (min)	40.0 (20.6)	26.2 (19.5)	19.0 (11.5)	15.2 (10.0)	10.6 (6.9)
$d_{\max}$ (min)	517 (186)	422 (207)	362 (188)	312 (166)	278 (156)
IS[b]	0.274 (0.15)	0.274 (0.15)	0.274 (0.15)	0.274 (0.15)	0.274 (0.15)
IV[b]	0.965 (0.307)	0.965 (0.307)	0.965 (0.307)	0.965 (0.307)	0.965 (0.307)
RA[b]	0.674 (0.209)	0.674 (0.209)	0.674 (0.209)	0.674 (0.209)	0.674 (0.209)
L5[b]	16.4 (15.9)	16.4 (15.9)	16.4 (15.9)	16.4 (15.9)	16.4 (15.9)
M10[b]	98.7 (88.8)	98.7 (88.8)	98.7 (88.8)	98.7 (88.8)	98.7 (88.8)

[a] here a power-law model is compared to a lognormal model, hence, a negative sign indicates a better fit of the lognormal model; [b] the circadian parameters are by construction independent of the considered time resolutions

Table 3: Results from multiple linear regressions at different time resolutions using the minimum and maximum durations of low-activity intervals used in the fitting procedure as predictors for the scaling exponents of the fitted power-laws. Both minimum and maximum durations were highly significant predictors for all considered time resolutions.

	CPM	CP30s	CP15s	CP10s	CP5s
$F(2,33)$	39.106	27.206	24.902	38.934	54.419
p	<0.001	<0.001	<0.001	<0.001	<0.001
Adjusted R^2	0.685	0.600	0.577	0.684	0.753

Table 4: The same as Table 2 excluding the three participants with day-night-reversal.

Variable	CPM	CP30s	CP15s	CP10s	CP5s
α_1	0.983 (0.077)	0.946 (0.078)	0.939 (0.078)	0.937 (0.078)	0.937 (0.079)
α_2	0.902 (0.146)	0.902 (0.148)	0.902 (0.150)	0.903 (0.150)	0.902 (0.150)
α_{12}	0.081 (0.160)	0.044 (0.164)	0.037 (0.166)	0.034 (0.166)	0.035 (0.166)
$\lvert\alpha_{12}\rvert$	0.143 (0.106)	0.137 (0.098)	0.138 (0.097)	0.137 (0.097)	0.138 (0.096)
LLR (obtained using $\boldsymbol{d}_{\min}^{LN}$)	1.831 (0.900)	2.270 (1.006)	2.670 (0.924)	2.385 (1.297)	3.219 (1.537)
LLR[a] (obtained using $\boldsymbol{d}_{\min}^{PL}$)	-0.569 (0.674)	-0.832 (0.740)	-0.857 (0.741)	-0.676 (0.684)	-0.733 (0.942)
n	19 (58%)	25 (76%)	27 (82%)	23 (70%)	30 (91%)
GOF ratio	1.405 (0.474)	1.368 (0.377)	1.414 (0.458)	1.477 (0.625)	1.409 (0.543)
γ	1.340 (0.533)	1.253 (0.440)	1.214 (0.532)	1.316 (0.662)	1.232 (0.542)
$d_{\min}^{PL}$ (min)	31.9 (29.7)	18.3 (17.0)	11.5 (15.8)	11.3 (14.0)	6.7 (9.4)
$d_{\min}^{LN}$ (min)	39.8 (21.2)	24.6 (19.0)	18.6 (10.9)	15.5 (10.3)	10.7 (7.1)
$d_{\max}$ (min)	514 (177)	425 (203)	362 (182)	314 (165)	280 (158)
IS[b]	0.292 (0.143)	0.292 (0.143)	0.292 (0.143)	0.292 (0.143)	0.292 (0.143)
IV[b]	0.966 (0.321)	0.966 (0.321)	0.966 (0.321)	0.966 (0.321)	0.966 (0.321)
RA[b]	0.706 (0.182)	0.706 (0.182)	0.706 (0.182)	0.706 (0.182)	0.706 (0.182)
L5[b]	15.2 (14.2)	15.2 (14.2)	15.2 (14.2)	15.2 (14.2)	15.2 (14.2)
M10[b]	103.2 (90.8)	103.2 (90.8)	103.2 (90.8)	103.2 (90.8)	103.2 (90.8)

[a] here a power-law model is compared to a lognormal model, hence, a negative sign indicates a better fit of the lognormal model; [b] the circadian parameters are by construction independent of the considered time resolutions

3 Dependence of measures of fractal characteristics on time resolution, gender, mobility and presence of day-night-reversal, falls and physical restraining

In the tables of this section, we supply sample means and bias-accelerated bootstrapped 95%-CIs for the quantities $|\alpha_{12}|$, LLR and GOF (see Section 2 of the main article for details) for all considered time resolutions, i.e. counts-per-minute (CPM), counts-per-30-seconds (CP30s), counts-per-15-seconds (CP15s), counts-per-10-seconds (CP10s) and counts-per-5-seconds (CP5s).

Table 5: Sample means and bias-accelerated bootstrapped 95%-CIs for $|\alpha_{12}|$, LLR and GOF for the 36 study participants.

	$\lvert\alpha_{12}\rvert$		LLR		GOF ratio	
	mean	95%-CI	mean	95%-CI	mean	95%-CI
CPM	0.141	[0.109, 0.177]	1.93	[1.64, 2.20]	1.435	[1.288, 1.589]
CP30s	0.136	[0.106, 0.169]	2.28	[1.97, 2.58]	1.387	[1.265, 1.509]
CP15s	0.137	[0.108, 0.169]	2.68	[2.37, 2.99]	1.424	[1.291, 1.555]
CP10s	0.135	[0.106, 0.168]	2.47	[2.06, 2.86]	1.485	[1.302, 1.679]
CP5s	0.136	[0.107, 0.169]	3.25	[2.78, 3.75]	1.436	[1.279, 1.608]

Table 6: The same as Table 5 excluding the three participants with day-night-reversal.

	$\lvert\alpha_{12}\rvert$		LLR		GOF ratio	
	mean	95%-CI	mean	95%-CI	mean	95%-CI
CPM	0.143	[0.108, 0.184]	1.83	[1.53, 2.11]	1.405	[1.258, 1.572]
CP30s	0.137	[0.104, 0.173]	2.27	[1.94, 2.59]	1.368	[1.248, 1.484]
CP15s	0.138	[0.106, 0.172]	2.67	[2.35, 2.96]	1.414	[1.266, 1.550]
CP10s	0.137	[0.105, 0.171]	2.39	[1.99, 2.77]	1.477	[1.294, 1.661]
CP5s	0.138	[0.107, 0.172]	3.22	[2.68, 3.69]	1.409	[1.225, 1.586]

Table 7: Sample means and bias-accelerated bootstrapped 95%-CIs for $|\alpha_{12}|$, LLR and GOF for the 17 male study participants.

	$\lvert\alpha_{12}\rvert$		LLR		GOF ratio	
	mean	95%-CI	mean	95%-CI	mean	95%-CI
CPM	0.127	[0.085, 0.170]	1.92	[1.56, 2.31]	1.457	[1.228, 1.710]
CP30s	0.121	[0.073, 0.171]	2.48	[1.96, 3.05]	1.411	[1.236, 1.618]
CP15s	0.124	[0.077, 0.173]	2.65	[2.21, 3.13]	1.450	[1.268, 1.649]
CP10s	0.122	[0.075, 0.171]	2.31	[1.84, 2.78]	1.481	[1.248, 1.741]
CP5s	0.123	[0.078, 0.172]	3.34	[2.55, 4.14]	1.432	[1.187, 1.679]

Table 8: Sample means and bias-accelerated bootstrapped 95%-CIs for $|\alpha_{12}|$, LLR and GOF for the 19 female study participants.

	$|\alpha_{12}|$		LLR		GOF ratio	
	mean	95%-CI	mean	95%-CI	mean	95%-CI
CPM	0.153	[0.103, 0.204]	1.94	[1.34, 2.42]	1.415	[1.247, 1.589]
CP30s	0.149	[0.103, 0.199]	2.10	[1.69, 2.52]	1.366	[1.199, 1.545]
CP15s	0.150	[0.104, 0.198]	2.71	[2.30, 3.11]	1.401	[1.175, 1.635]
CP10s	0.147	[0.100, 0.196]	2.61	[1.91, 3.33]	1.488	[1.214, 1.810]
CP5s	0.148	[0.102, 0.196]	3.17	[2.61, 3.72]	1.440	[1.213, 1.695]

Table 9: Sample means and bias-accelerated bootstrapped 95%-CIs for $|\alpha_{12}|$, LLR and GOF for the 16 autonomously mobile study participants.

	$|\alpha_{12}|$		LLR		GOF ratio	
	mean	95%-CI	mean	95%-CI	mean	95%-CI
CPM	0.155	[0.110, 0.199]	1.55	[1.01, 2.03]	1.342	[1.188, 1.491]
CP30s	0.141	[0.100, 0.180]	2.03	[1.57, 2.46]	1.322	[1.133, 1.524]
CP15s	0.143	[0.102, 0.182]	2.44	[1.98, 2.90]	1.307	[1.114, 1.507]
CP10s	0.140	[0.100, 0.177]	2.30	[1.80, 2.80]	1.426	[1.155, 1.722]
CP5s	0.142	[0.103, 0.180]	3.14	[2.32, 3.96]	1.344	[1.123, 1.574]

Table 10: Sample means and bias-accelerated bootstrapped 95%-CIs for $|\alpha_{12}|$, LLR and GOF for the 17 (at least partially) immobile study participants.

	$|\alpha_{12}|$		LLR		GOF ratio	
	mean	95%-CI	mean	95%-CI	mean	95%-CI
CPM	0.131	[0.075, 0.195]	2.18	[1.91, 2.47]	1.467	[1.202, 1.750]
CP30s	0.134	[0.087, 0.191]	2.42	[1.94, 2.95]	1.412	[1.259, 1.554]
CP15s	0.136	[0.088, 0.194]	2.94	[2.53, 3.35]	1.534	[1.323, 1.758]
CP10s	0.135	[0.087, 0.190]	2.49	[1.81, 3.14]	1.576	[1.287, 1.921]
CP5s	0.135	[0.090, 0.190]	3.37	[2.77, 3.94]	1.529	[1.264, 1.812]

Table 11: Sample means and bias-accelerated bootstrapped 95%-CIs for $|\alpha_{12}|$, LLR and GOF for the 22 study participants for who no fall was registered over their stay in hospital.

	$|\alpha_{12}|$		LLR		GOF ratio	
	mean	95%-CI	mean	95%-CI	mean	95%-CI
CPM	0.149	[0.111, 0.187]	2.01	[1.58, 2.41]	1.478	[1.283, 1.673]
CP30s	0.146	[0.112, 0.182]	2.42	[1.98, 2.87]	1.372	[1.190, 1.571]
CP15s	0.147	[0.114, 0.182]	2.63	[2.18, 3.06]	1.372	[1.189, 1.567]
CP10s	0.146	[0.114, 0.180]	2.41	[1.78, 3.04]	1.365	[1.154, 1.623]
CP5s	0.147	[0.114, 0.181]	3.04	[2.35, 3.72]	1.388	[1.164, 1.642]

Table 12: Sample means and bias-accelerated bootstrapped 95%-CIs for $|\alpha_{12}|$, LLR and GOF for the 14 study participants who fell at least one time over their stay in hospital according to the records taken by the clinical staff.

	$\|\alpha_{12}\|$		LLR		GOF ratio	
	mean	95%-CI	mean	95%-CI	mean	95%-CI
CPM	0.127	[0.074, 0.189]	1.80	[1.40, 2.18]	1.369	[1.194, 1.542]
CP30s	0.120	[0.073, 0.172]	2.06	[1.57, 2.56]	1.412	[1.266, 1.548]
CP15s	0.118	[0.076, 0.174]	2.76	[2.21, 3.28]	1.505	[1.278, 1.722]
CP10s	0.120	[0.072, 0.171]	2.57	[2.02, 3.07]	1.674	[1.383, 1.959]
CP5s	0.120	[0.075, 0.170]	3.58	[2.96, 4.34]	1.511	[1.268, 1.751]

Table 13: Sample means and bias-accelerated bootstrapped 95%-CIs for $|\alpha_{12}|$, LLR and GOF for the 21 study participants who were never subject to physical restraining over their stay in hospital.

	$\|\alpha_{12}\|$		LLR		GOF ratio	
	mean	95%-CI	mean	95%-CI	mean	95%-CI
CPM	0.176	[0.124, 0.227]	1.86	[1.32, 2.37]	1.439	[1.206, 1.704]
CP30s	0.162	[0.119, 0.209]	2.39	[1.86, 2.94]	1.363	[1.189, 1.569]
CP15s	0.159	[0.118, 0.205]	2.89	[2.45, 3.33]	1.377	[1.198, 1.580]
CP10s	0.157	[0.117, 0.201]	2.57	[1.89, 3.30]	1.402	[1.147, 1.717]
CP5s	0.158	[0.116, 0.201]	3.17	[2.48, 3.84]	1.313	[1.117, 1.539]

Table 14: Sample means and bias-accelerated bootstrapped 95%-CIs for $|\alpha_{12}|$, LLR and GOF for the 15 study participants who were subject to physical restraining at least once over their stay in hospital according to the records of the clinical staff.

	$\|\alpha_{12}\|$		LLR		GOF ratio	
	mean	95%-CI	mean	95%-CI	mean	95%-CI
CPM	0.091	[0.058, 0.128]	2.03	[1.70, 2.38]	1.429	[1.255, 1.631]
CP30s	0.099	[0.064, 0.138]	2.13	[1.65, 2.55]	1.422	[1.244, 1.585]
CP15s	0.106	[0.070, 0.146]	2.38	[1.81, 2.93]	1.489	[1.271, 1.688]
CP10s	0.105	[0.069, 0.145]	2.32	[1.74, 2.90]	1.601	[1.386, 1.818]
CP5s	0.107	[0.069, 0.148]	3.37	[2.65, 4.12]	1.608	[1.348, 1.880]

4 Correlation tables

In Tables 15-18, we provide Pearson correlation coefficients and their bias-accelerated bootstrapped 95%-CIs for pair-wise associations between the variables α_1, α_2, α_{12} and $|\alpha_{12}|$ resulting from the five used time resolutions CPM, CP30s, CP15s, CP10s and CP5s. In Tables 19 and 20, we provide the same for the log-likelihood-ratios (LLR) and the GOF ratios. See Sections 2.2.1 and 2.2.2 of the main article for a description of the mentioned quantities. Correlation coefficients are shown above the diagonal and CIs below the diagonal in Tables 15-19.

Table 15: Correlational analysis for α_1 obtained via DFA of all participants' activity data.

	CPM	CP30S	CP15S	CP10S	CP5S
CPM	1	0.995	0.994	0.994	0.994
CP30S	[0.991, 0.997]	1	0.999	0.998	0.997
CP15S	[0.990, 0.997]	[0.998, 0.999]	1	0.999	0.999
CP10S	[0.990, 0.997]	[0.996, 0.999]	[0.998, 1.0]	1	0.999
CP5S	[0.989, 0.997]	[0.995, 0.999]	[0.998, 0.999]	[0.998, 0.999]	1

Table 16: Correlational analysis for α_2 obtained via DFA of all participants' activity data.

	CPM	CP30S	CP15S	CP10S	CP5S
CPM	1	0.996	0.996	0.996	0.997
CP30S	[0.988, 0.999]	1	0.997	0.997	0.997
CP15S	[0.986, 0.999]	[0.994, 0.999]	1	0.998	0.999
CP10S	[0.991, 0.999]	[0.994, 0.999]	[0.995, 1.0]	1	>0.999
CP5S	[0.993, 0.999]	[0992., 0.999]	[0.997, 1.0]	[0.999, 1.0]	1

Table 17: Correlational analysis for α_{12} obtained via DFA of all participants' activity data.

	CPM	CP30S	CP15S	CP10S	CP5S
CPM	1	0.996	0.996	0.997	0.997
CP30S	[0.993, 0.998]	1	0.997	0.998	0.998
CP15S	[0.990, 0.999]	[0.993, 0.999]	1	0.998	0.999
CP10S	[0.995, 0.999]	[0.996, 0.999]	[0.996, 1.0]	1	0.999
CP5S	[0.994, 0.998]	[0.997, 0.999]	[0.997, 1.0]	[0.999, 1.0]	1

In Table 21 we provide pair-wise correlation coefficients and bias-accelerated bootstrapped CIs for associations between the LLRs at the five used time resolutions (CPM, CP30s, CP15s, CP10s and CP5s) and the considered circadian parameters, the age and the MMSE scores of the study participants. In Table 22, we provide the same for the GOF ratios. Note that MMSE scores were available only for 31 out of 36 study participants.

Table 18: Associations (and bias-accelerated bootstrapped CIs) between the LLRs obtained via analysis of all participants' CDDs (based on $d_{\min}^{\mathrm{LN}}$, see Section 2.2.1 of the main article) and the variables IS, IV, RA, M10, the age and the MMSE scores of the study participants. Note that MMSE scores were available only for 31 out of 36 study participants.

	IS	IV	RA	M10	Age	MMSE
CPM	-0.244	0.066	-0.149	-0.096	0.286	-0.003
	[-0.573,0.244]	[-0.203,0.322]	[-0.436,0.234]	[-0.384,0.293]	[-0.088,0.593]	[-0.338, 0.404]
CP30s	-0.207	-0.020	-0.025	0.014	0.223	0.142
	[-0.574,0.291]	[-0.318,0.252]	[-0.347,0.315]	[-0.440,0.471]	[-0.119,0.502]	[-0.205, 0.488]
CP15s	-0.140	0.060	-0.043	-0.152	0.037	0.265
	[-0.520,0.352]	[-0.271,0.383]	[-0.366,0.308]	[-0.475,0.326]	[-0.244,0.324]	[-0.140, 0.603]
CP10s	-0.090	0.163	-0.077	-0.237	-0.021	0.148
	[-0.412,0.323]	[-0.131,0.443]	[-0.397,0.292]	[-0.544,0.133]	[-0.371,0.325]	[-0.227, 0.484]
CP5s	-0.058	-0.128	-0.029	0.113	-0.129	0.129
	[-0.398,0.378]	[-0.392,0.146]	[-0.445,0.448]	[-0.229,0.423]	[-0.328,0.139]	[-0.231, 0.443]

Table 19: Associations (and bias-accelerated bootstrapped CIs) between the GOF ratios obtained via analysis of all participants' CDDs (based on $d_{\min}^{\mathrm{LN}}$, see Section 2.2.1 of the main article) and the variables IS, IV, RA, M10, the age and the MMSE scores of the study participants. Note that MMSE scores were available only for 31 out of 36 study participants.

	IS	IV	RA	M10	Age	MMSE
CPM	-0.155	-0.028	-0.153	-0.051	0.182	-0.091
	[-0.444,0.126]	[-0.360,0.327]	[-0.385,0.065]	[-0.352,0.277]	[-0.242,0.551]	[-0.491,0.291]
CP30s	-0.233	-0.120	0.015	-0.078	-0.027	-0.015
	[-0.524,0.147]	[-0.358,0.118]	[-0.339,0.342]	[-0.429,0.332]	[-0.436,0.391]	[-0.431,0.361]
CP15s	-0.264	0.109	-0.141	-0.076	0.006	0.161
	[-0.530,0.052]	[-0.469,0.230]	[-0.479,0.209]	[-0.428,0.258]	[-0.342,0.337]	[-0.243,0.500]
CP10s	-0.237	0.028	-0.153	-0.072	0.006	0.153
	[-0.498,0.011]	[-0.425,0.311]	[-0.471,0.132]	[-0.379,0.271]	[-0.348,0.389]	[-0.200,0.456]
CP5s	-0.220	-0.174	-0.220	-0.096	-0.065	-0.149
	[-0.475,0.068]	[-0.452,0.135]	[-0.562,0.138]	[-0.346,0.167]	[-0.407,0.265]	[-0.485,0.256]

Table 20: Computed pair-wise correlation coefficients (above diagonal) and bootstrapped 95%-CIs (below diagonal) for the DFA parameters α_1, α_2, α_{12}, $|\alpha_{12}|$ (obtained using the time resolution CP15s) of all study participants except those three with day-night reversal..

	α_1	α_2	α_{12}	$\|\alpha_{12}\|$
α_1	1	0.035	0.438	0.246
α_2	[-0.333,0.340]	1	-0.883	-0.172
α_{12}	[0.098,0.722]	[-0.936,-0.801]	1	0.271
$\|\alpha_{12}\|$	[-0.111,0.587]	[-0.625,0.256]	[-0.261,0.768]	1

Table 21: Associations (and bias-accelerated bootstrapped CIs) between the DFA parameters α_1, α_2, α_{12}, $|\alpha_{12}|$ obtained using the time resolution CP15s and the variables IS, IV, RA, M10, the age and the MMSE scores of all study participants except those three with day-night reversal. Note that MMSE scores were available only for 28 out of 33 participants.

	IS	IV	RA	M10	Age	MMSE
α_1	0.439<br>[0.163,0.665]	0.130<br>[-0.249,0.471]	0.201<br>[-0.139,0.531]	0.583<br>[0.312,0.809]	-0.030<br>[-0.369,0.294]	0.491<br>[0.110,0.773]
α_2	0.280<br>[-0.061,0.565]	-0.885<br>[-0.944,-0.802]	0.123<br>[-0.159,0.387]	0.528<br>[0.179,0.726]	-0.135<br>[-0.410,0.130]	-0.347<br>[-0.608,-0.052]
α_{12}	-0.046<br>[-0.460,0.355]	0.858<br>[0.764,0.917]	-0.016<br>[-0.317,0.285]	-0.202<br>[-0.547,0.287]	0.108<br>[-0.149,0.356]	0.547<br>[0.276,0.752]
$\|\alpha_{12}\|$	0.333<br>[-0.056,0.645]	0.408<br>[0.002,0.765]	0.257<br>[-0.104,0.562]	0.180<br>[-0.195,0.498]	-0.146<br>[-0.425,0.143]	0.214<br>[-0.169,0.545]

Table 22: Computed pair-wise correlation coefficients (above diagonal) and bias-accelerated bootstrapped 95%-CIs (below diagonal) for the circadian parameters IS, IV, RA, M10, the age (in years), and the MMSE scores of the participants except those three with day-night-reversal. Note that MMSE scores were available only for 28 out of 33 participants.

	IS	IV	RA	M10	Age	MMSE
IS	1	-0.04	0.727	0.644	-0.218	-0.082
IV	[-0.378,0.331]	1	0.019	-0.366	0.144	0.429
RA	[0.506,0.889]	[-0.378,0.345]	1	0.352	-0.321	-0.309
M10	[0.428,0.800]	[-0.624,0.037]	[0.056,0.583]	1	-0.242	0.085
Age	[-0.593,0.150]	[-0.057,0.365]	[-0.620,0.005]	[-0.548,0.049]	1	0.099
MMSE	[-0.484,0.371]	[0.132,0.666]	[-0.708,0.150]	[-0.317,0.572]	[-0.363,0.465]	1

5 Graphs of individual CDDs including PL and LN fits

In the following, we provide the obtained individual graphs of CDDs based on the time resolution of CP5s. They include also depictions of distribution functions of the power law (PL) and lognormal (LN) distributions which were obtained via fitting them to the empirical CDDs as described in Section 2 of the main article. Via visual inspection, we could separate the graphs into the following groups (and subgroups):

1. LN appears to fit the data considerably better than PL (29 instances)
    a. LN appears to fit the data considerably better than PL, however, only far in the tail of the empirical distribution, i.e. $d_{\mathrm{min}}^{\mathrm{LN}} \gg d_{\mathrm{min}}^{\mathrm{PL}}$ (seven instances)
    b. LN appears to fit the data considerably better than PL with $d_{\mathrm{min}}^{\mathrm{LN}} > d_{\mathrm{min}}^{\mathrm{PL}}$ (13 instances)
    c. LN appears to fit the data considerably better than PL with $d_{\mathrm{min}}^{\mathrm{LN}} \approx d_{\mathrm{min}}^{\mathrm{PL}}$ or $d_{\mathrm{min}}^{\mathrm{LN}} < d_{\mathrm{min}}^{\mathrm{PL}}$ (nine instances)
2. Both LN and PL appear to fit the data quite well (four instances)
3. Both LN and PL provide poor fits to the data (three instances)

In the following sections, we list the graphs of empirical CDDs for all participants according to those groups.

In Section 5.1, we list the graphs which correspond to group 1.a, i.e. those CDDs for which LN appears to fit the data considerably better than PL, however, only far in the tail of the empirical distribution, i.e. $d_{\mathrm{min}}^{\mathrm{LN}} \gg d_{\mathrm{min}}^{\mathrm{PL}}$. This was the case in 7 instances.

In Section 5.2, we list the graphs which correspond to group 1.b, i.e. those CDDs for which LN appears to fit the data considerably better than PL with $d_{\mathrm{min}}^{\mathrm{LN}} > d_{\mathrm{min}}^{\mathrm{PL}}$. This was the case in 13 instances.

In Section 5.3, we list the graphs which correspond to group 1.c, i.e. those CDDs for which LN appears to fit the data considerably better than PL with $d_{\mathrm{min}}^{\mathrm{LN}} \approx d_{\mathrm{min}}^{\mathrm{PL}}$ or $d_{\mathrm{min}}^{\mathrm{LN}} < d_{\mathrm{min}}^{\mathrm{PL}}$. This was the case in 9 instances.

In Section 5.4, we list the graphs which correspond to group 2, i.e. those CDDs for which both LN and PL appear to fit the data quite well. This was the case in four instances.

In Section 5.5, we list the graphs which correspond to group 2, i.e. those CDDs for which both LN and PL provide poor fits to the data. This was the case in three instances.

5.1 Empirical CDDs according to Group 1.a

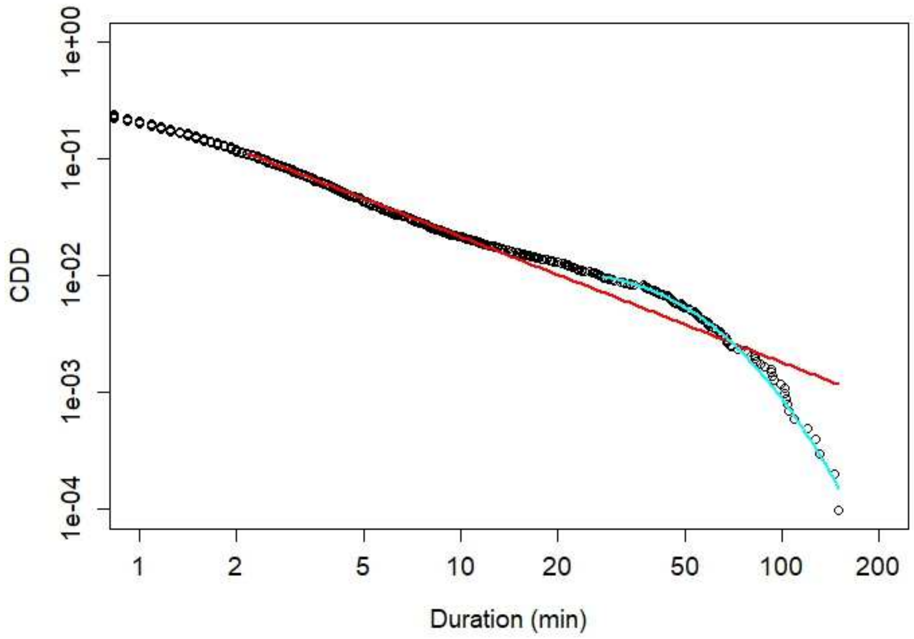

Figure 1: Empirical CDD (black circles) and best fitting PL (red line) and LN (cyan line) distributions of participant 1.

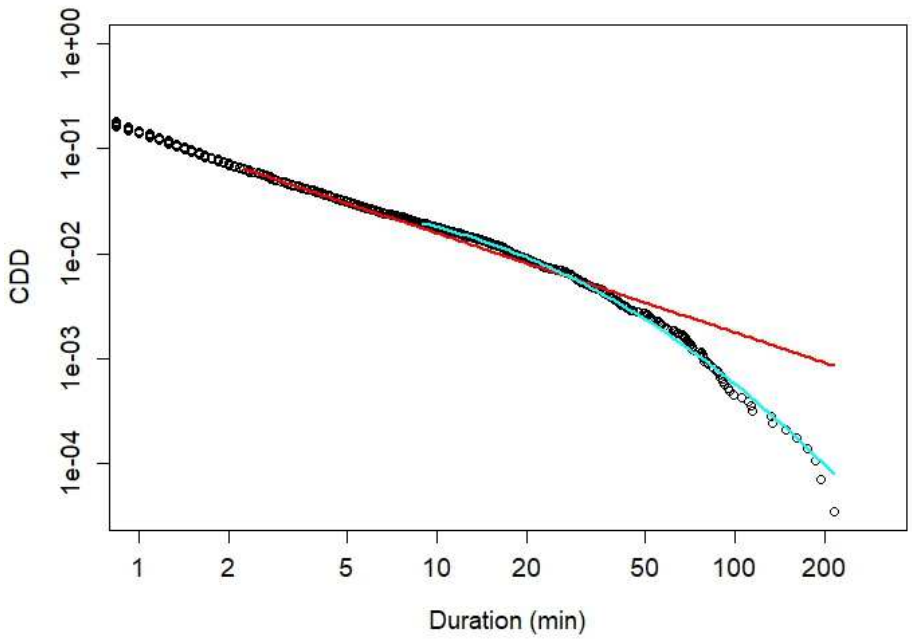

Figure 2: Empirical CDD (black circles) and best fitting PL (red line) and LN (cyan line) distributions of participant 8.

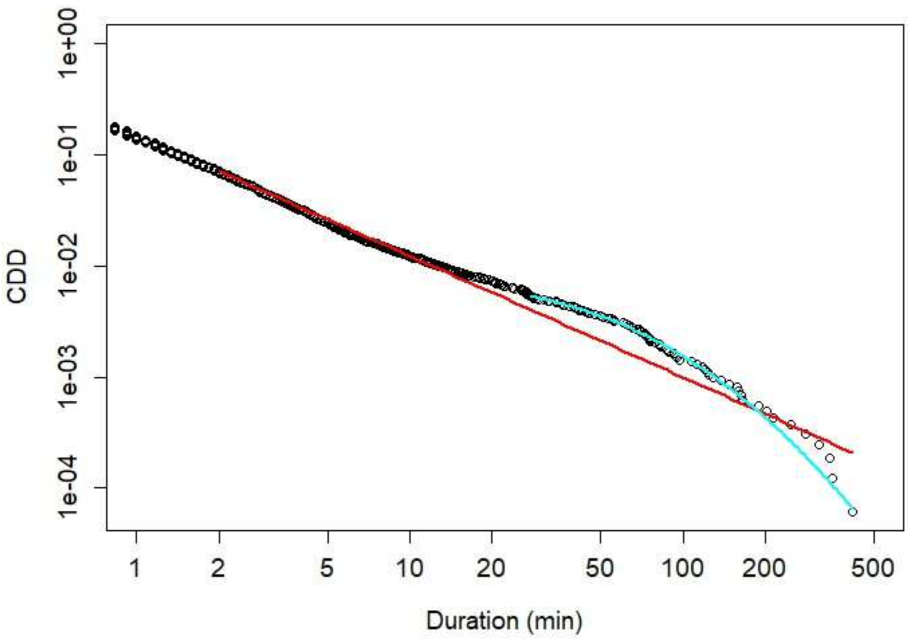

Figure 3: Empirical CDD (black circles) and best fitting PL (red line) and LN (cyan line) distributions of participant 12.

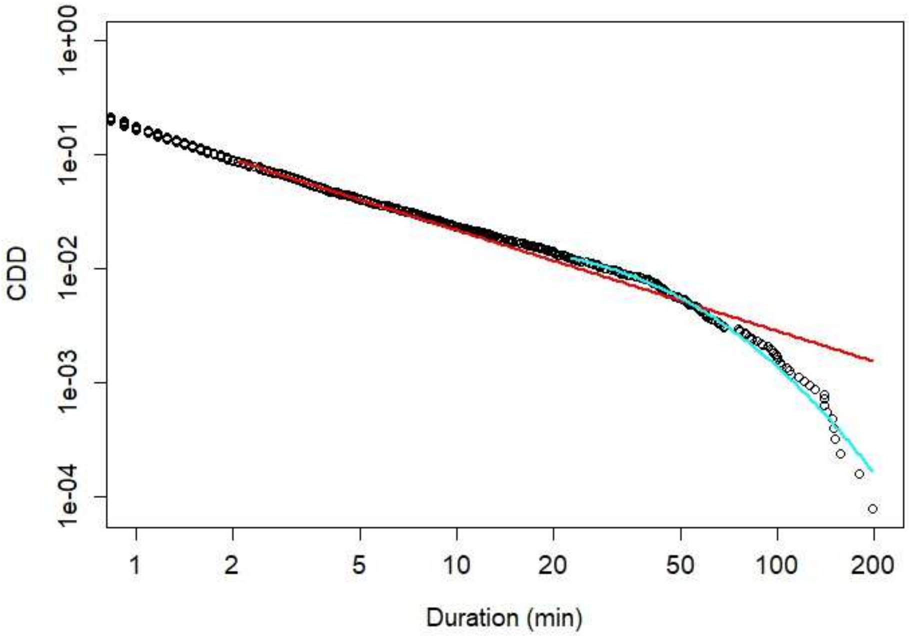

Figure 4: Empirical CDD (black circles) and best fitting PL (red line) and LN (cyan line) distributions of participant 22.

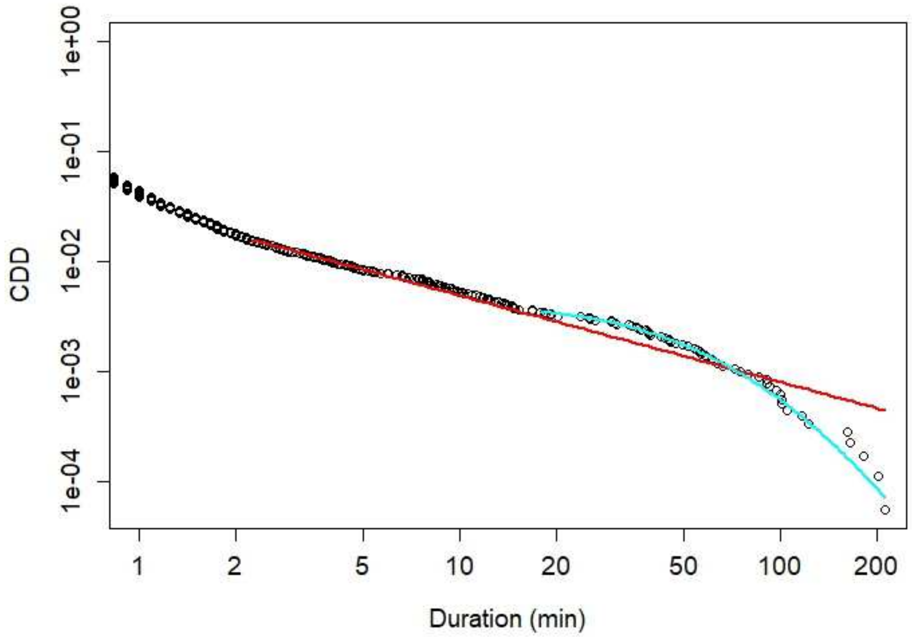

Figure 5: Empirical CDD (black circles) and best fitting PL (red line) and LN (cyan line) distributions of participant 24.

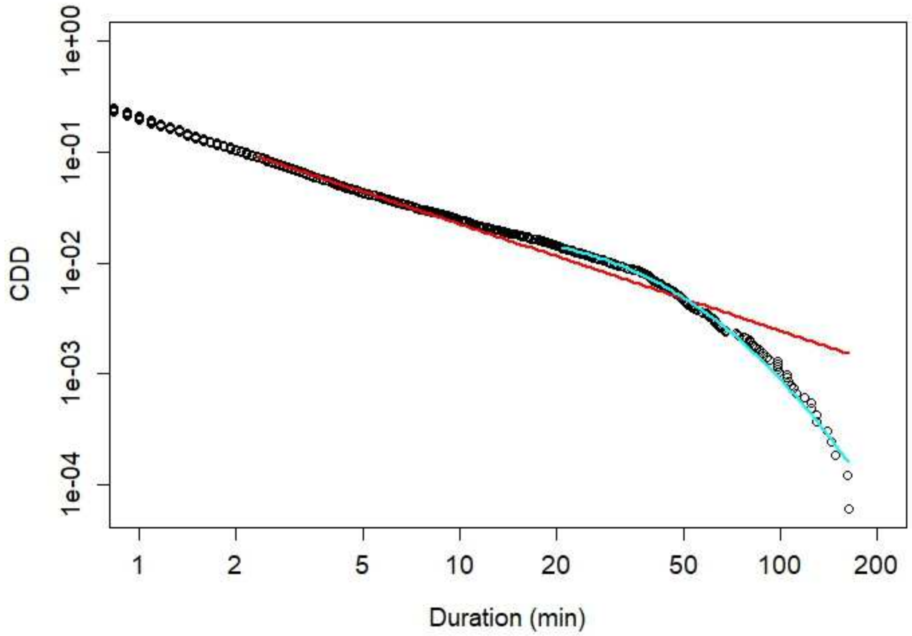

Figure 6: Empirical CDD (black circles) and best fitting PL (red line) and LN (cyan line) distributions of participant 27.

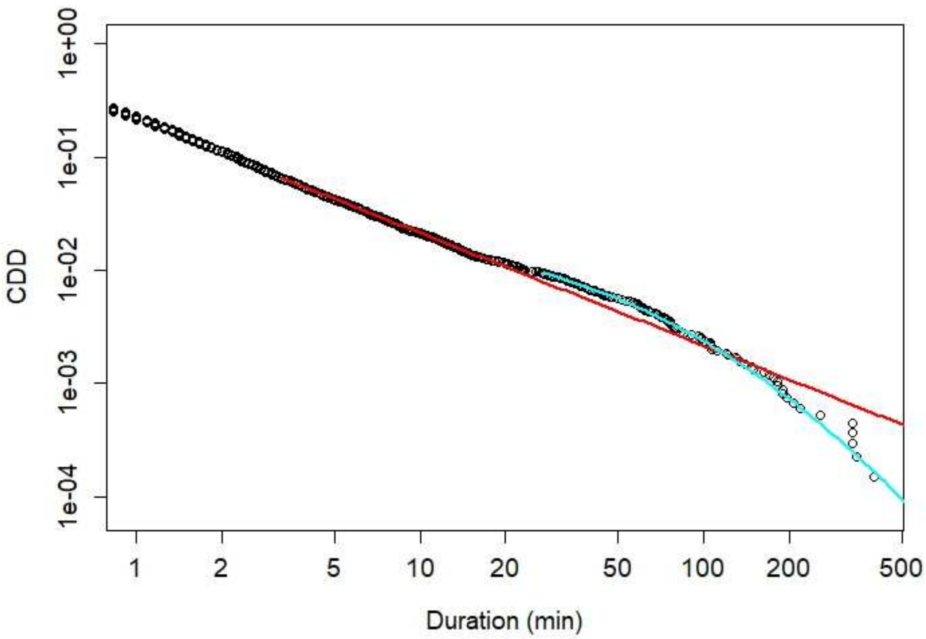

Figure 7: Empirical CDD (black circles) and best fitting PL (red line) and LN (cyan line) distributions of participant 34.

5.2 Empirical CDDs according to Group 1.b

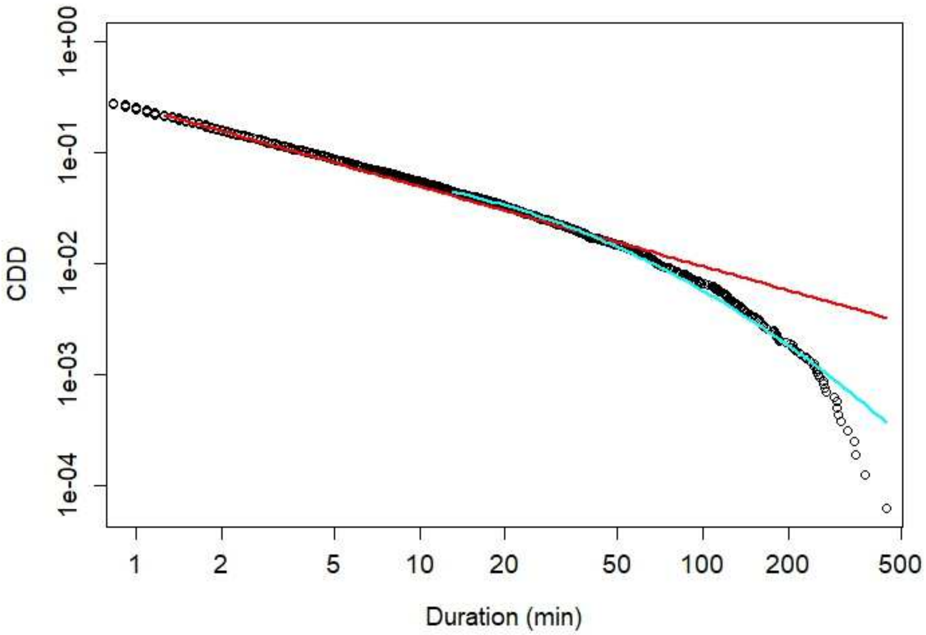

Figure 8: Empirical CDD (black circles) and best fitting PL (red line) and LN (cyan line) distributions of participant 2.

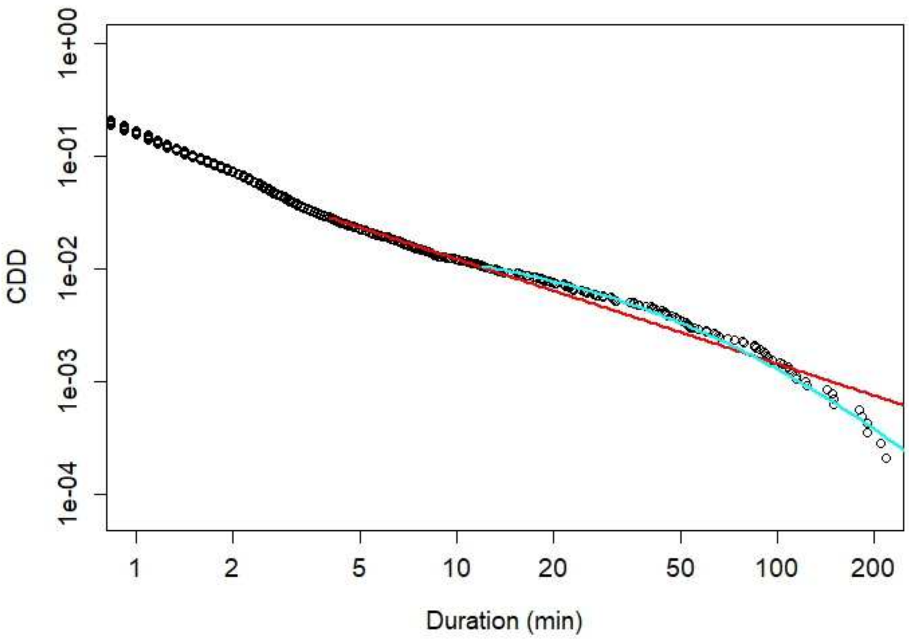

Figure 9: Empirical CDD (black circles) and best fitting PL (red line) and LN (cyan line) distributions of participant 14.

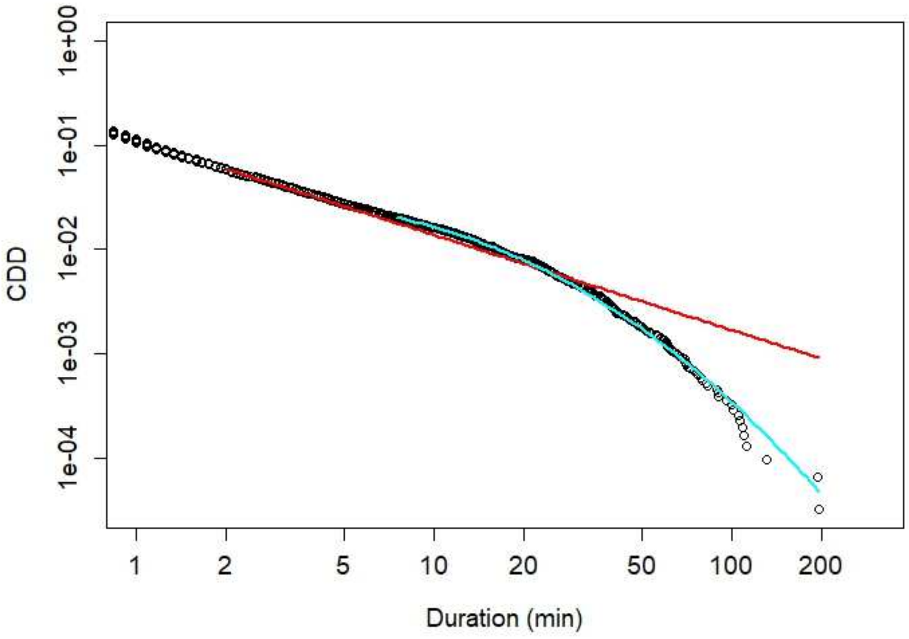

Figure 10: Empirical CDD (black circles) and best fitting PL (red line) and LN (cyan line) distributions of participant 16.

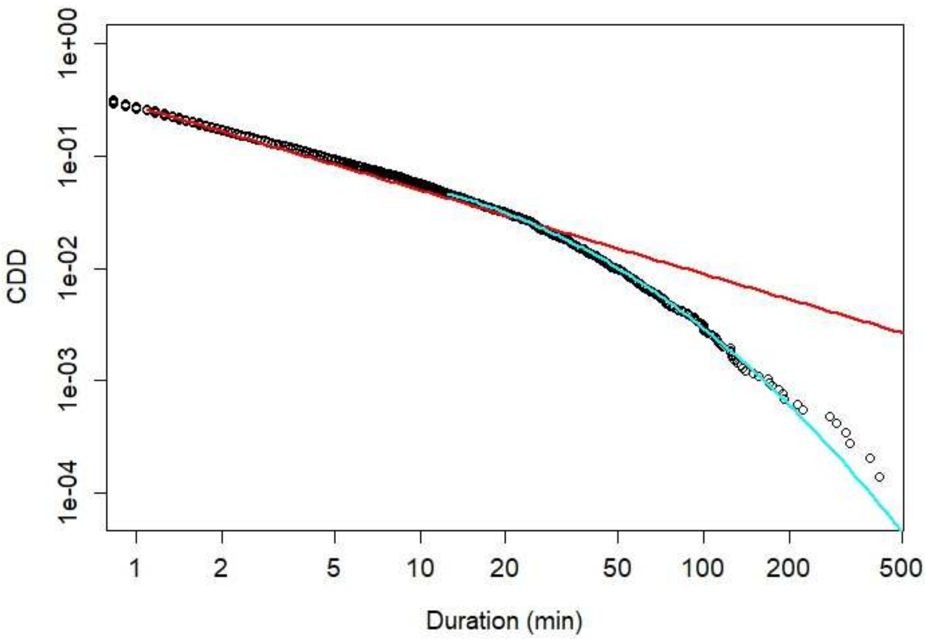

Figure 11: Empirical CDD (black circles) and best fitting PL (red line) and LN (cyan line) distributions of participant 17.

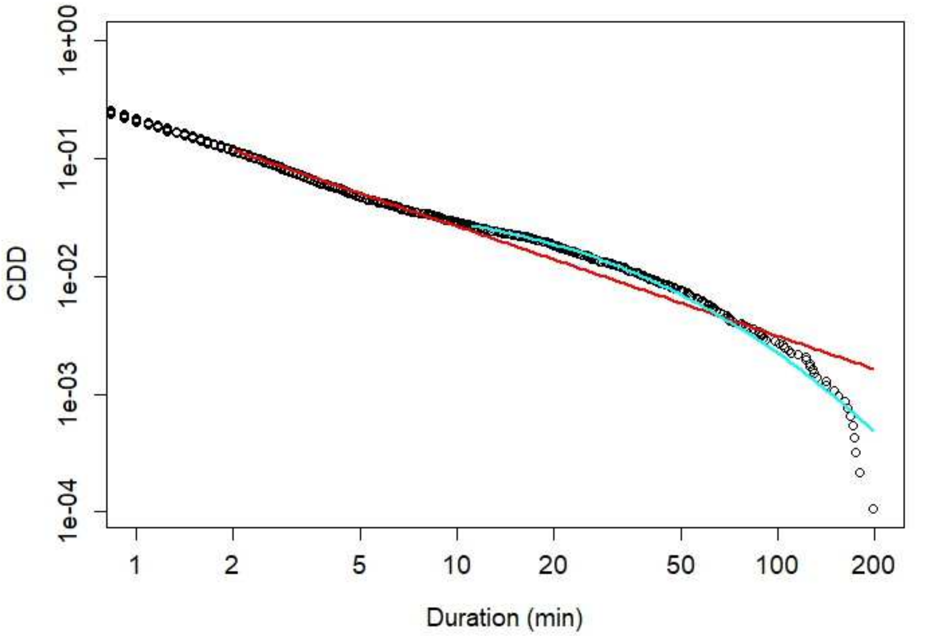

Figure 12: Empirical CDD (black circles) and best fitting PL (red line) and LN (cyan line) distributions of participant 19.

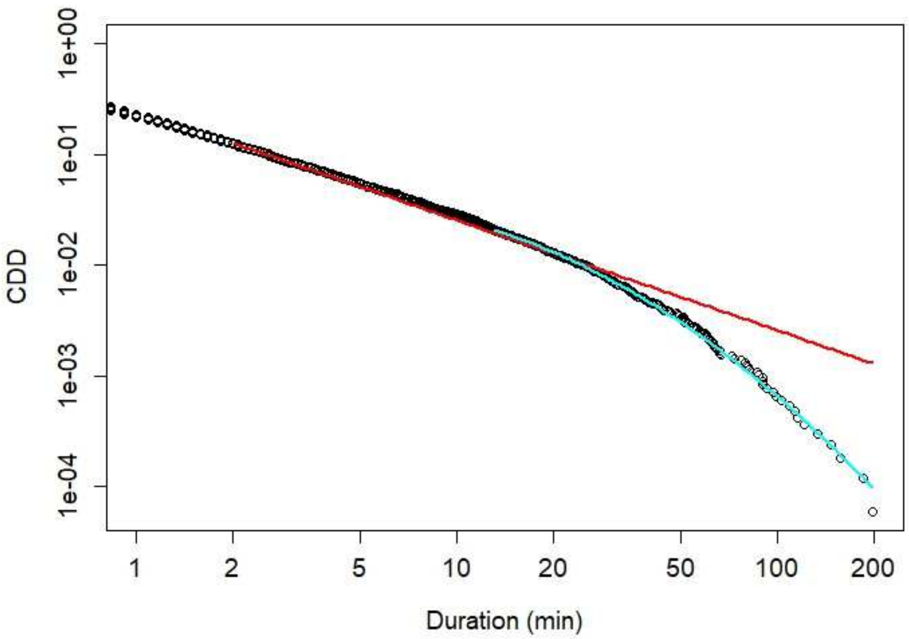

Figure 13: Empirical CDD (black circles) and best fitting PL (red line) and LN (cyan line) distributions of participant 20.

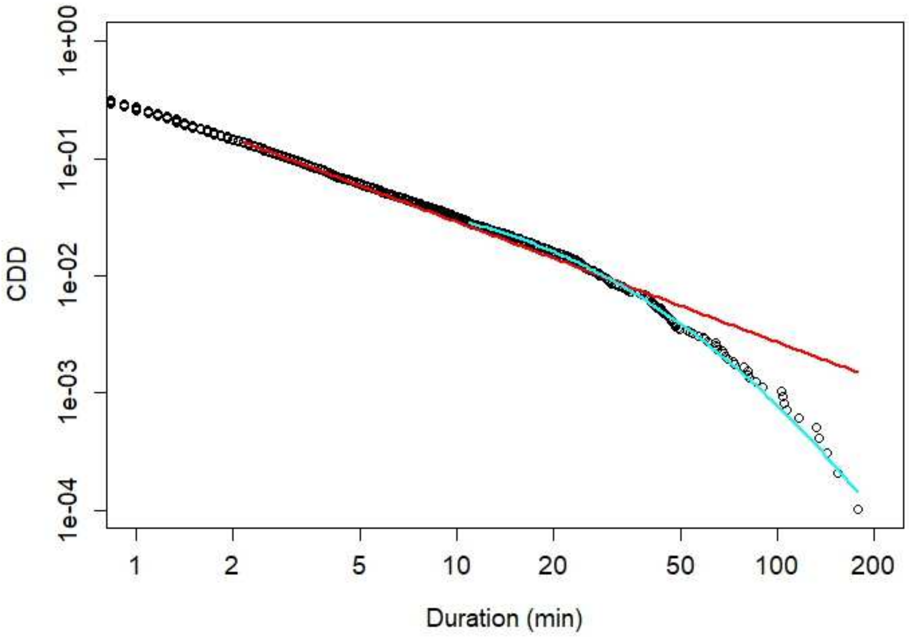

Figure 14: Empirical CDD (black circles) and best fitting PL (red line) and LN (cyan line) distributions of participant 25.

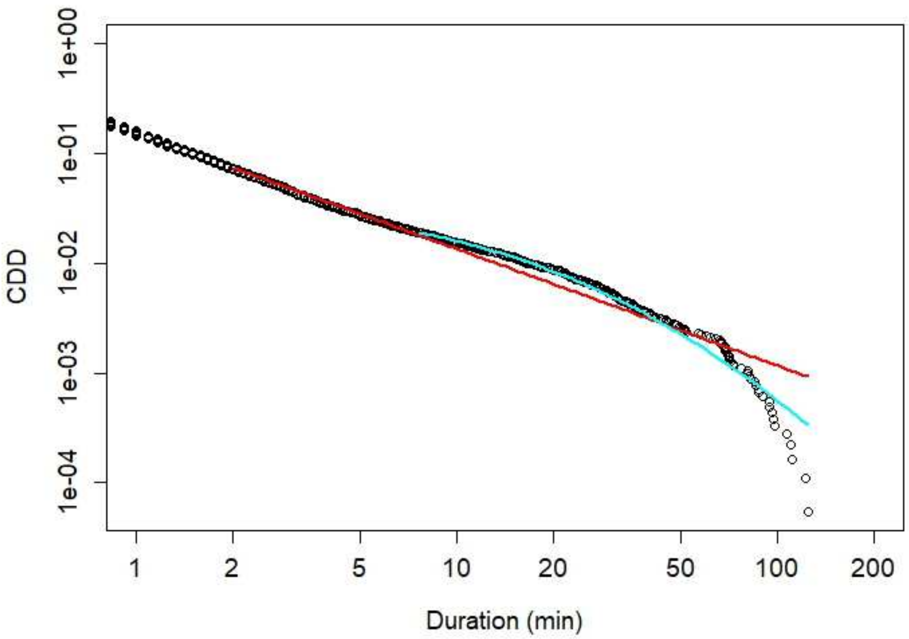

Figure 15: Empirical CDD (black circles) and best fitting PL (red line) and LN (cyan line) distributions of participant 28.

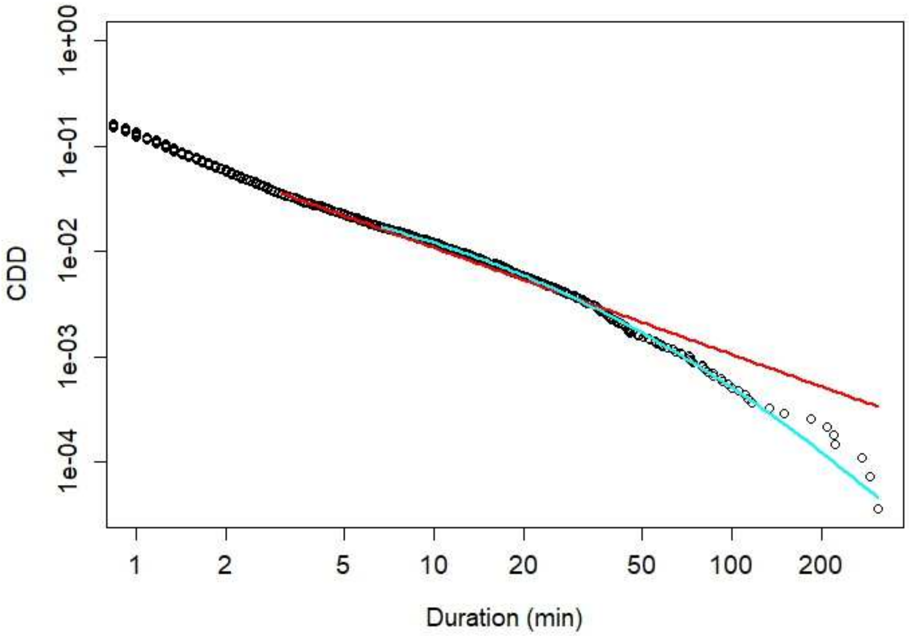

Figure 16: Empirical CDD (black circles) and best fitting PL (red line) and LN (cyan line) distributions of participant 29.

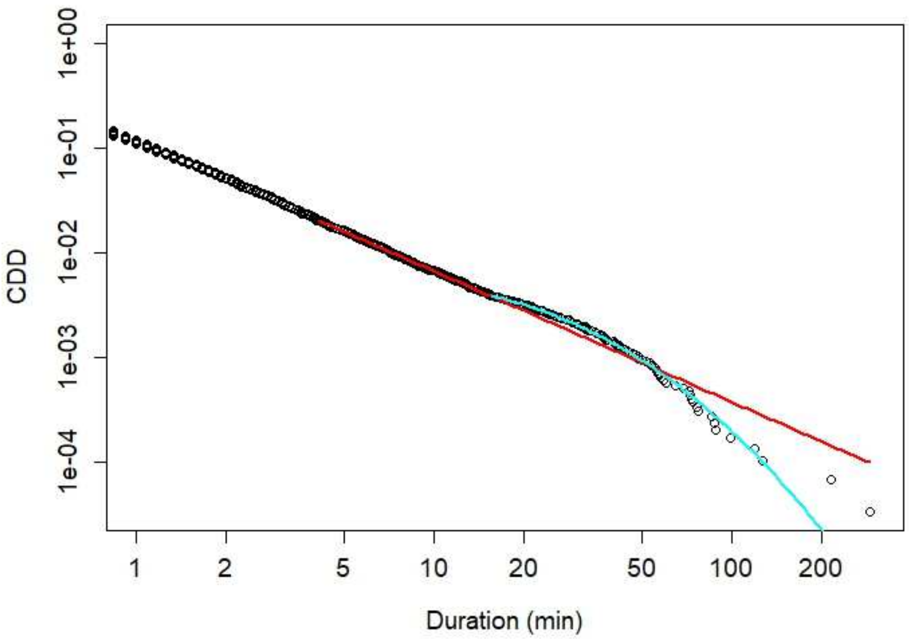

Figure 17: Empirical CDD (black circles) and best fitting PL (red line) and LN (cyan line) distributions of participant 30.

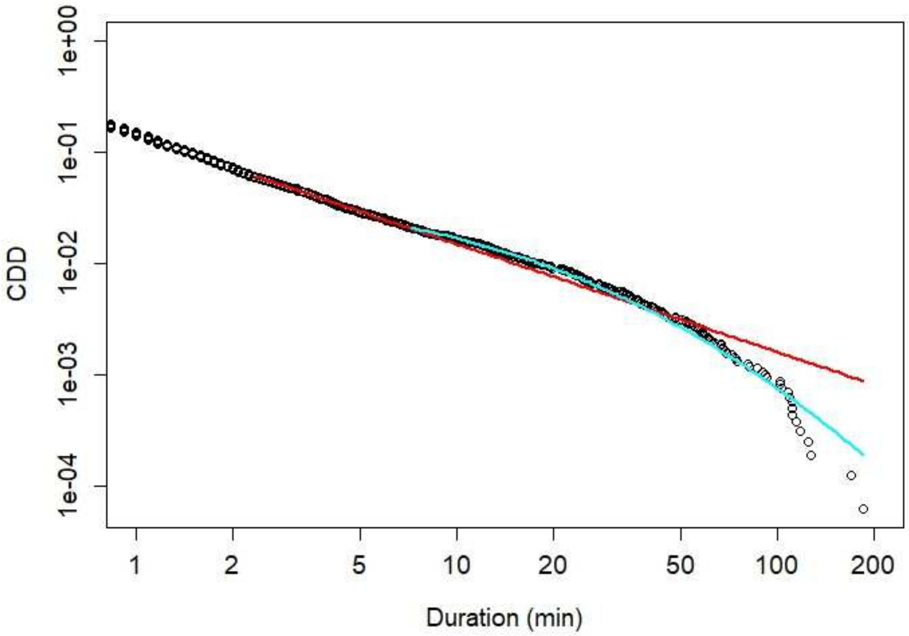

Figure 18: Empirical CDD (black circles) and best fitting PL (red line) and LN (cyan line) distributions of participant 33.

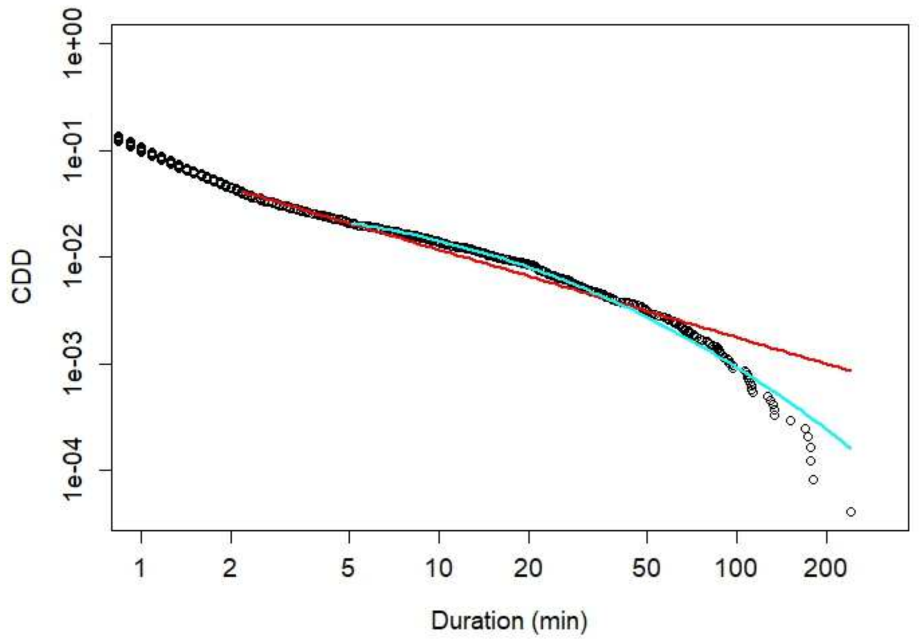

Figure 19: Empirical CDD (black circles) and best fitting PL (red line) and LN (cyan line) distributions of participant 35.

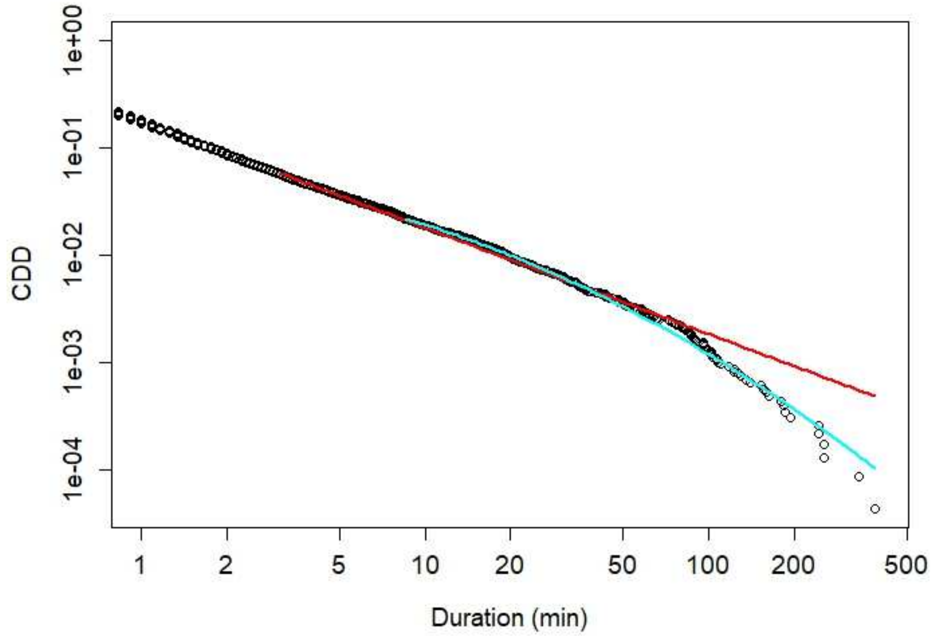

Figure 20: Empirical CDD (black circles) and best fitting PL (red line) and LN (cyan line) distributions of participant 36.

5.3 Empirical CDDs according to Group 1.c

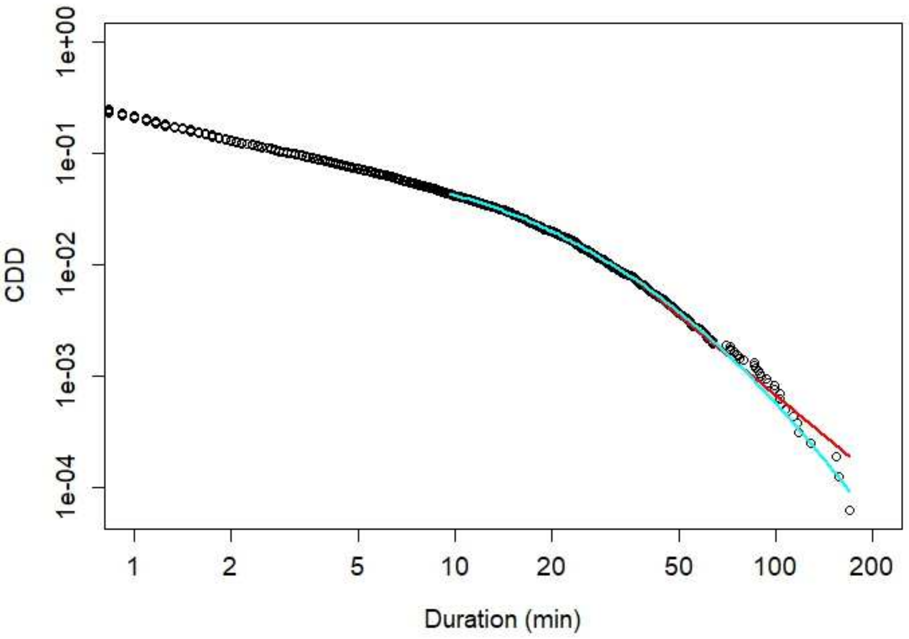

Figure 21: Empirical CDD (black circles) and best fitting PL (red line) and LN (cyan line) distributions of participant 4.

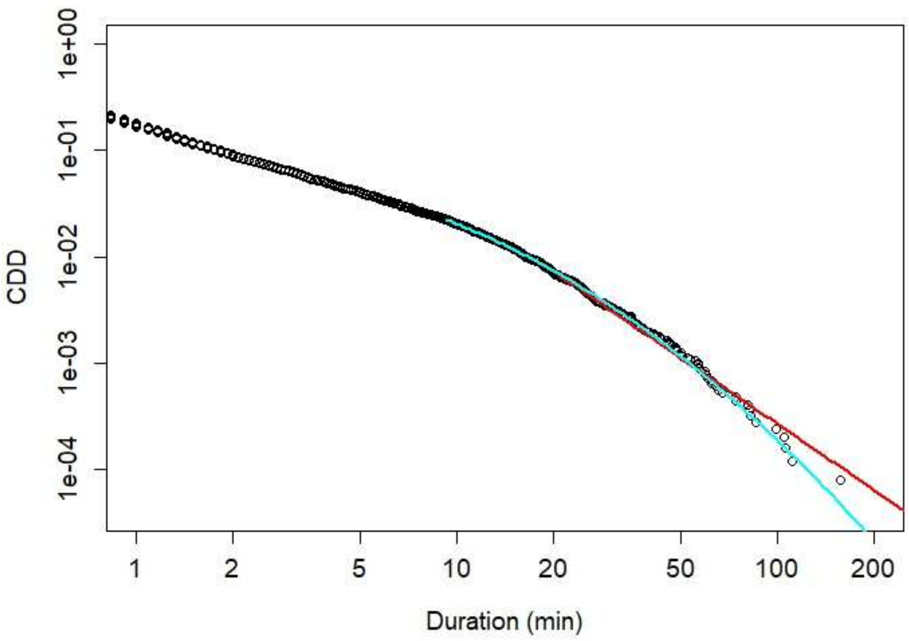

Figure 22: Empirical CDD (black circles) and best fitting PL (red line) and LN (cyan line) distributions of participant 5.

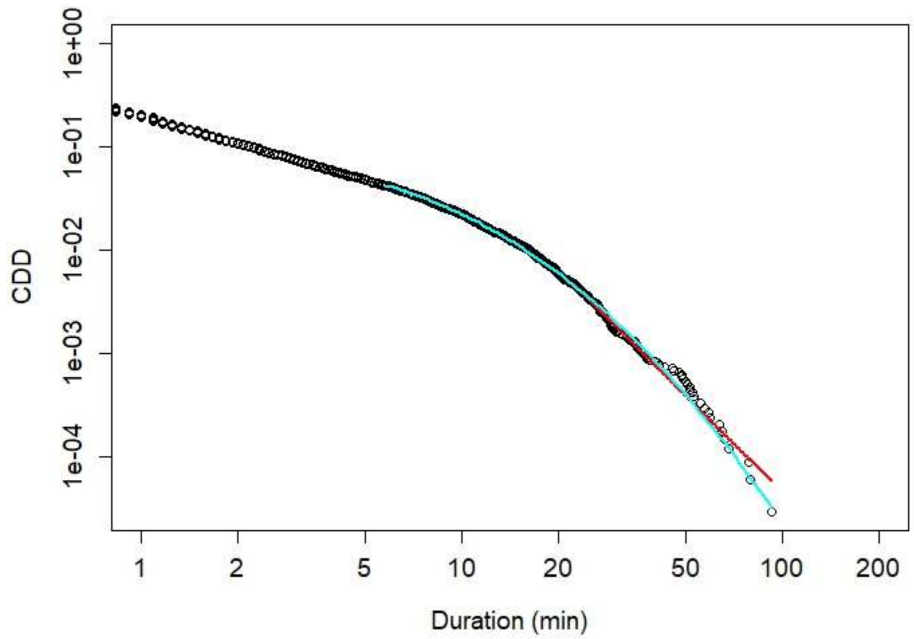

Figure 23: Empirical CDD (black circles) and best fitting PL (red line) and LN (cyan line) distributions of participant 6.

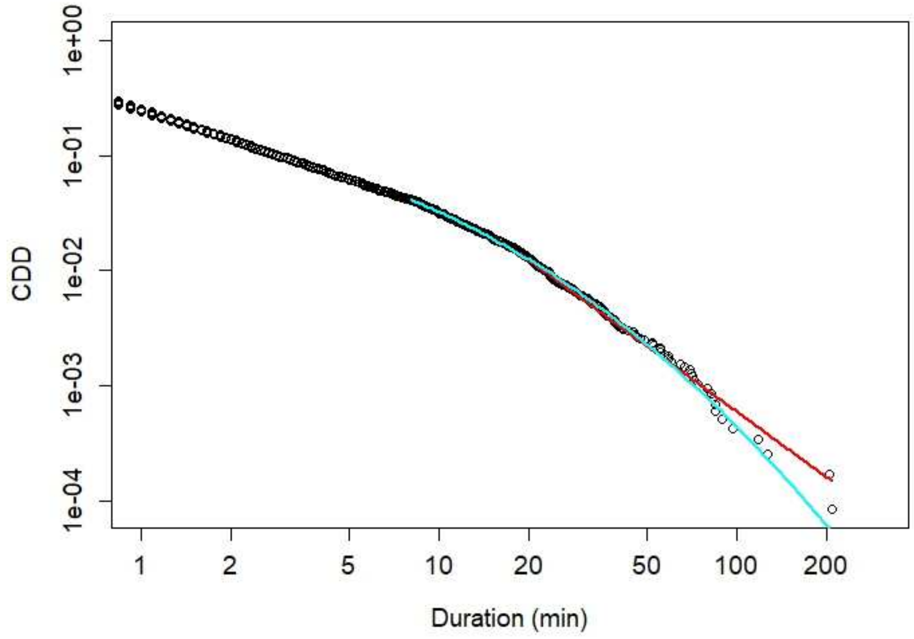

Figure 24: Empirical CDD (black circles) and best fitting PL (red line) and LN (cyan line) distributions of participant 9.

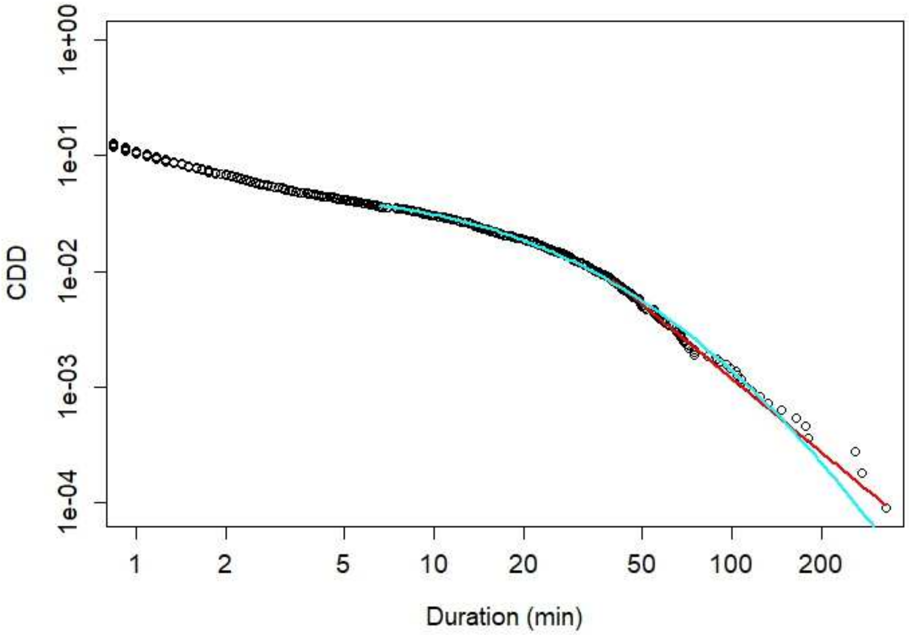

Figure 25: Empirical CDD (black circles) and best fitting PL (red line) and LN (cyan line) distributions of participant 11.

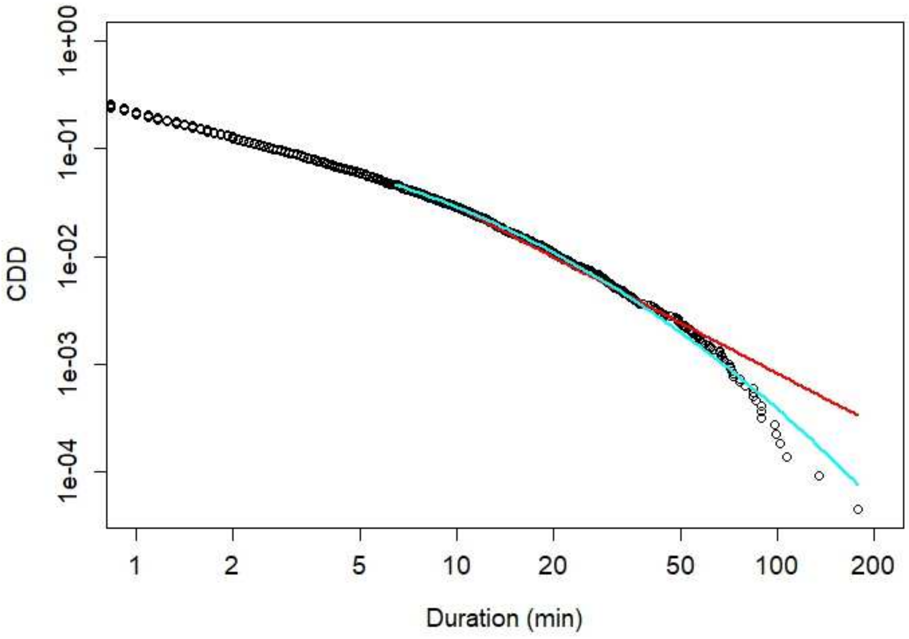

Figure 26: Empirical CDD (black circles) and best fitting PL (red line) and LN (cyan line) distributions of participant 21.

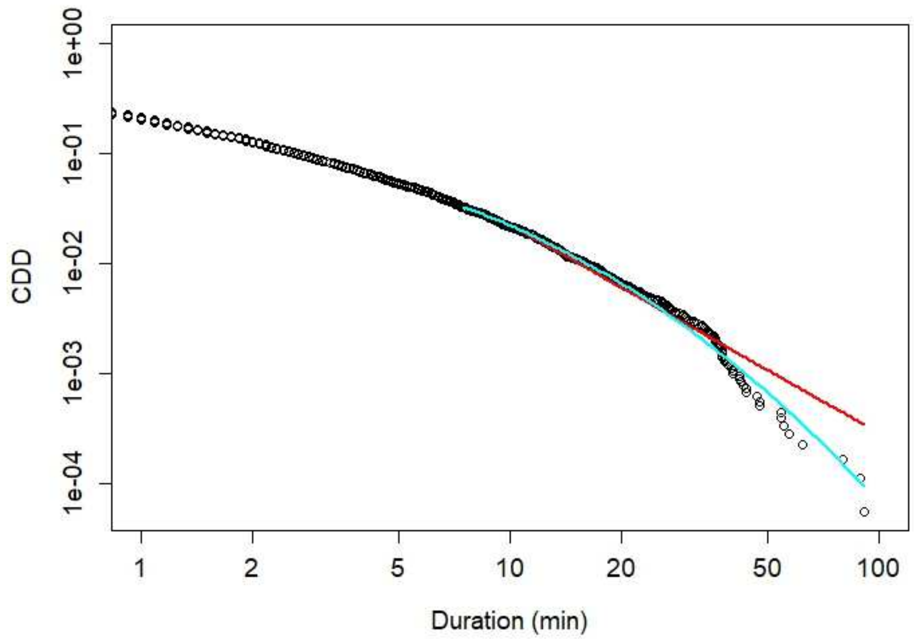

Figure 27: Empirical CDD (black circles) and best fitting PL (red line) and LN (cyan line) distributions of participant 23.

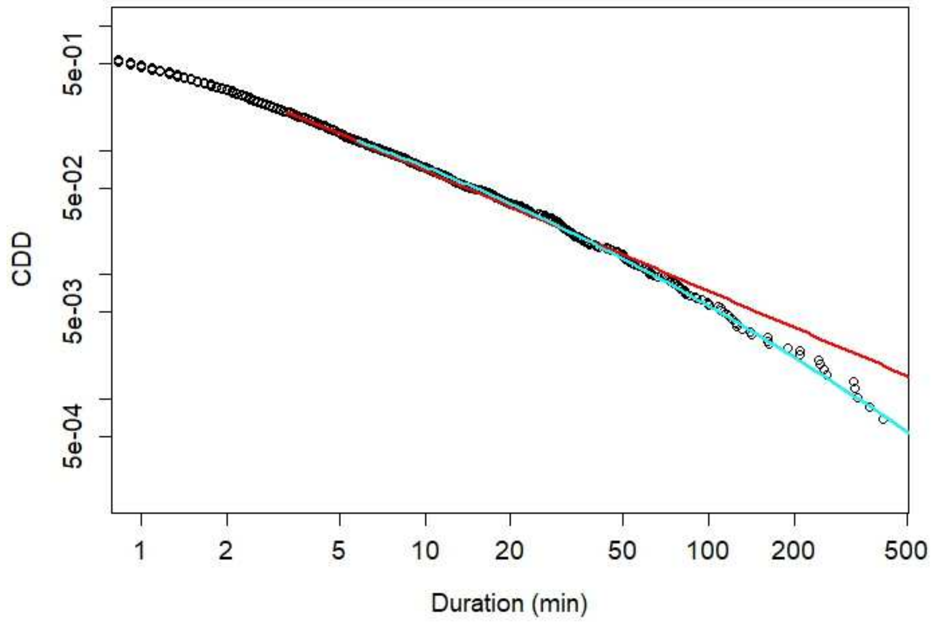

Figure 28: Empirical CDD (black circles) and best fitting PL (red line) and LN (cyan line) distributions of participant 26.

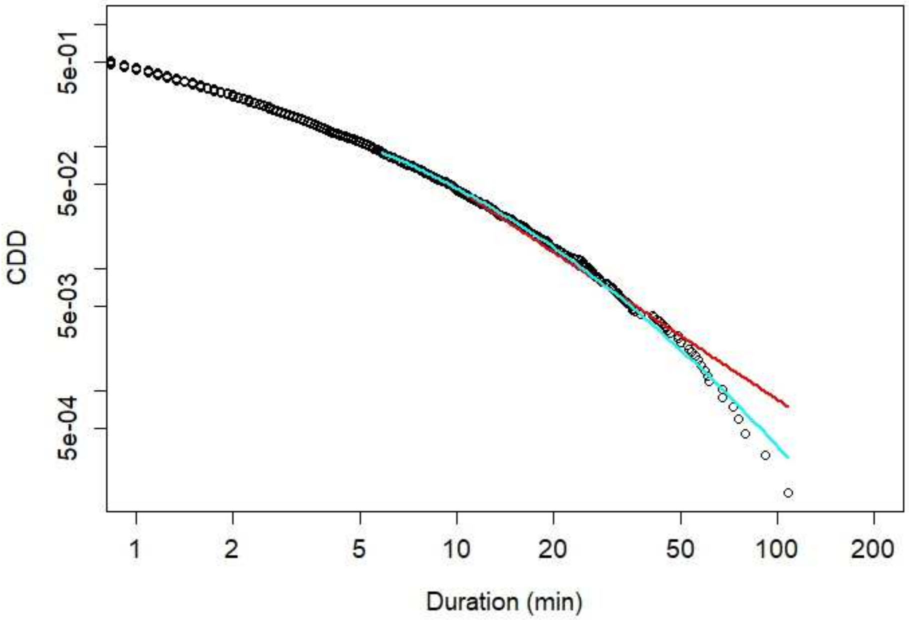

Figure 29: Empirical CDD (black circles) and best fitting PL (red line) and LN (cyan line) distributions of participant 31.

5.4 Empirical CDDs according to Group 2

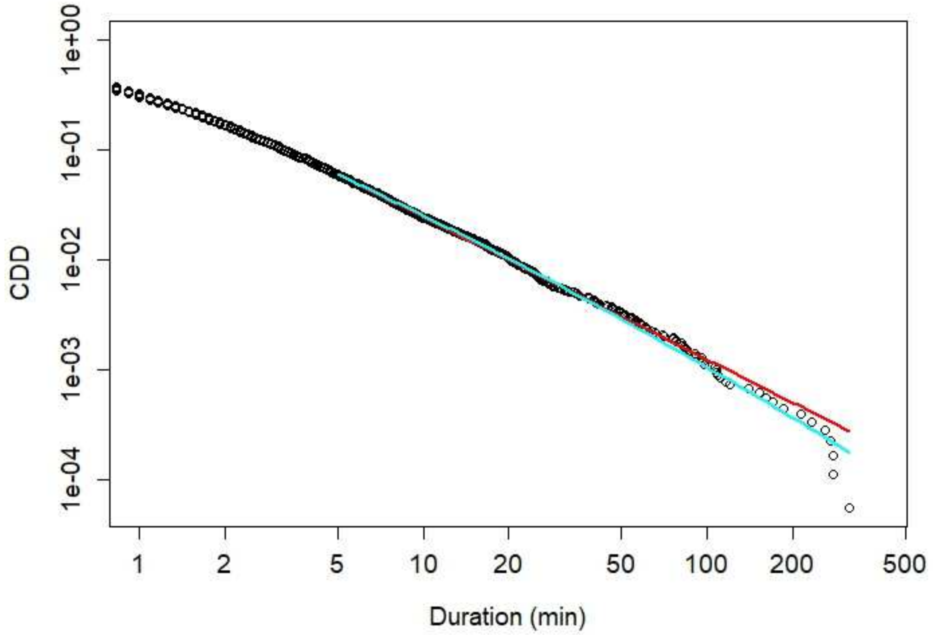

Figure 30: Empirical CDD (black circles) and best fitting PL (red line) and LN (cyan line) distributions of participant 3.

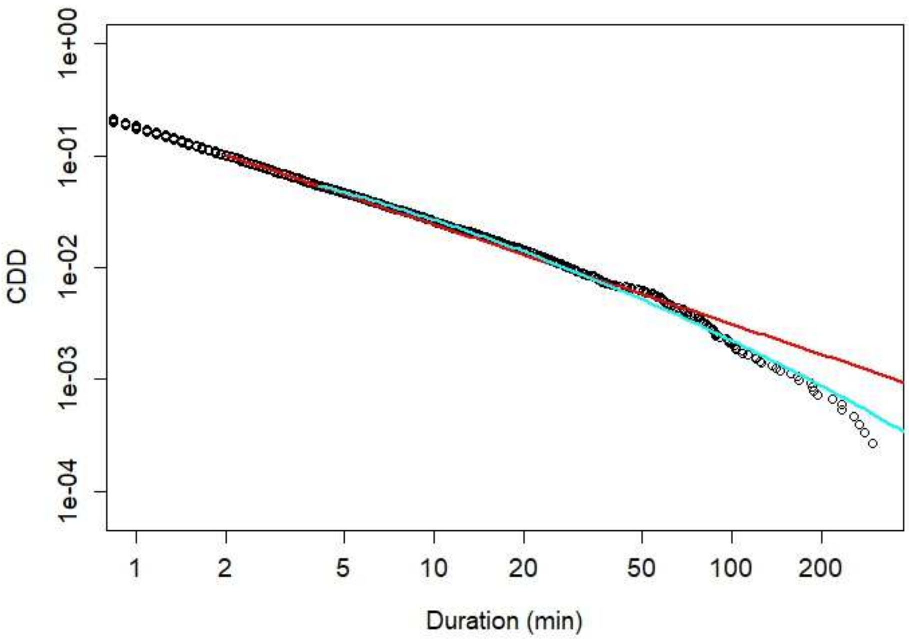

Figure 31: Empirical CDD (black circles) and best fitting PL (red line) and LN (cyan line) distributions of participant 7.

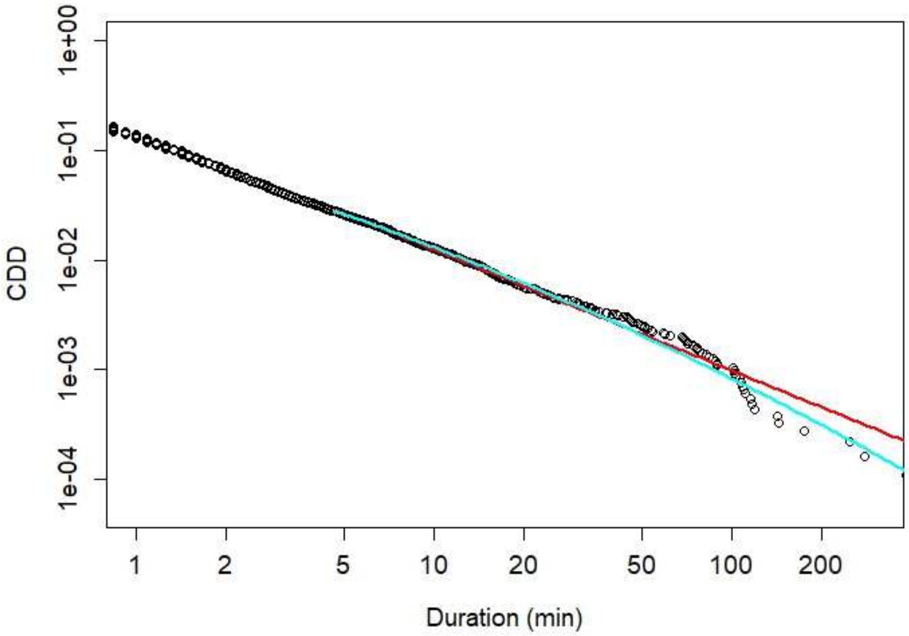

Figure 32: Empirical CDD (black circles) and best fitting PL (red line) and LN (cyan line) distributions of participant 10.

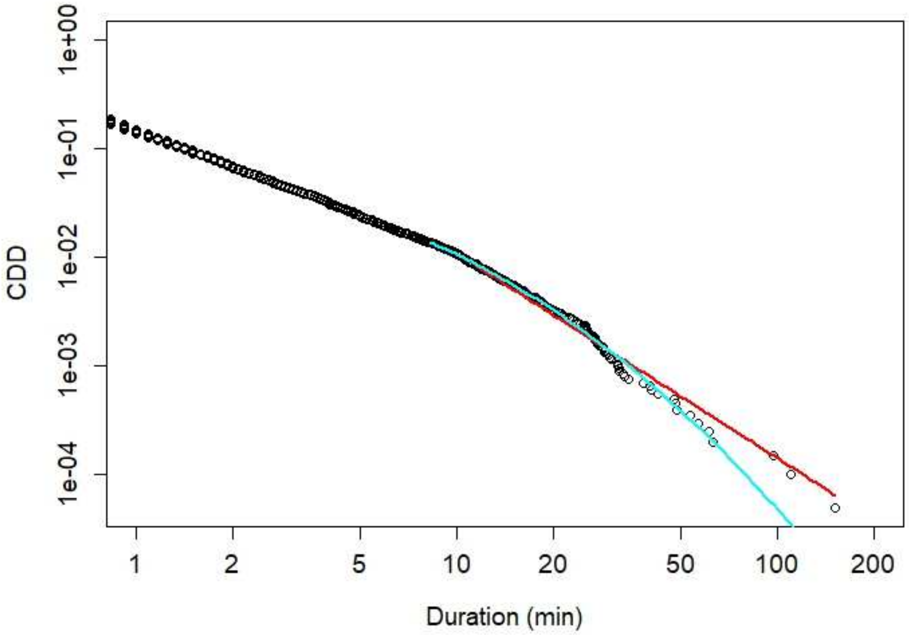

Figure 33: Empirical CDD (black circles) and best fitting PL (red line) and LN (cyan line) distributions of participant 13.

5.5 Empirical CDDs according to Group 3

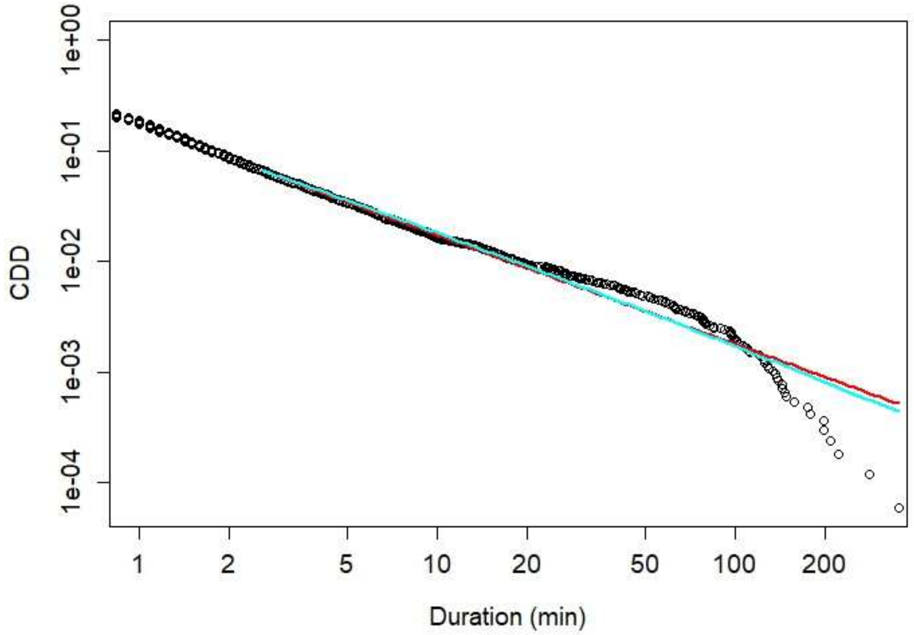

Figure 34: Empirical CDD (black circles) and best fitting PL (red line) and LN (cyan line) distributions of participant 15.

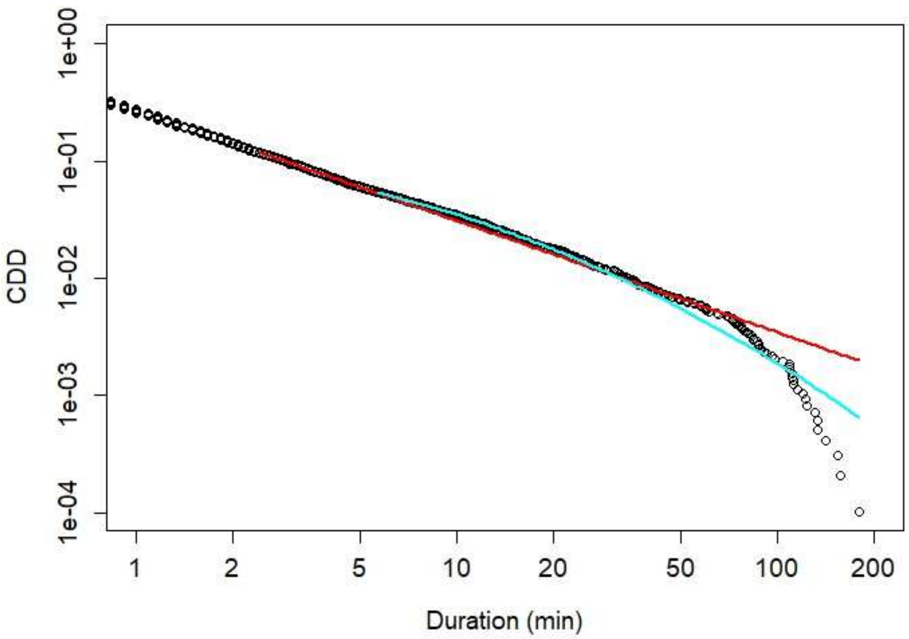

Figure 35: Empirical CDD (black circles) and best fitting PL (red line) and LN (cyan line) distributions of participant 18.

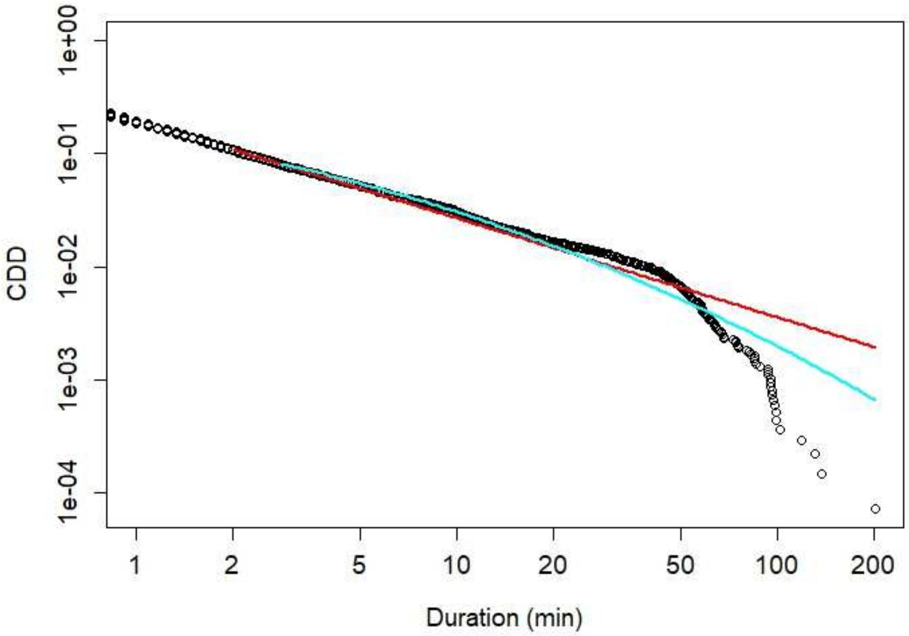

Figure 36: Empirical CDD (black circles) and best fitting PL (red line) and LN (cyan line) distributions of participant 32.
